# Longitudinal Associations of Blood Phosphorylated Tau181 and Neurofilament Light Chain With Neurodegeneration in Alzheimer Disease

**DOI:** 10.1001/jamaneurol.2020.4986

**Published:** 2021-01-11

**Authors:** Alexis Moscoso, Michel J. Grothe, Nicholas J. Ashton, Thomas K. Karikari, Juan Lantero Rodríguez, Anniina Snellman, Marc Suárez-Calvet, Kaj Blennow, Henrik Zetterberg, Michael Schöll

**Affiliations:** 1 Department of Psychiatry and Neurochemistry, Institute of Neuroscience and Physiology, The Sahlgrenska Academy, University of Gothenburg, Gothenburg, Sweden; 2 Wallenberg Centre for Molecular and Translational Medicine, University of Gothenburg, Gothenburg, Sweden; 3 Unidad de Trastornos del Movimiento, Instituto de Biomedicina de Sevilla, Hospital Universitario Virgen del Rocío/CSIC/Universidad de Sevilla, Seville, Spain; 4 King’s College London, Institute of Psychiatry, Psychology & Neuroscience, Maurice Wohl Clinical Neuroscience Institute, London, United Kingdom; 5 NIHR Biomedical Research Centre for Mental Health & Biomedical Research Unit for Dementia at South London & Maudsley NHS Foundation, London, United Kingdom; 6 Turku PET Centre, University of Turku, Turku, Finland; 7 Barcelonaßeta Brain Research Center, Pasqual Maragall Foundation. Barcelona, Spain; 8 Hospital del Mar Medical Research Institute, Barcelona, Spain; 9 Servei de Neurologia, Hospital del Mar, Barcelona, Spain; 10 Centro de Investigación Biomédica en Red de Fragilidad y Envejecimiento Saludable, Madrid, Spain; 11 Clinical Neurochemistry Laboratory, Sahlgrenska University Hospital, Mölndal, Sweden; 12 Department of Neurodegenerative Disease, UCL Queen Square Institute of Neurology, University College London, London, United Kingdom; 13 UK Dementia Research Institute at University College London, London, United Kingdom

## Abstract

**Importance:**

Plasma phosphorylated tau at threonine 181 (p-tau181) has been proposed as an easily accessible biomarker for the detection of Alzheimer disease (AD) pathology, but its ability to monitor disease progression in AD remains unclear.

**Objective:**

To study the potential of longitudinal plasma p-tau181 measures for assessing neurodegeneration progression and cognitive decline in AD in comparison to plasma neurofilament light chain (NfL), a disease-nonspecific marker of neuronal injury.

**Design, Setting, and Participants:**

This longitudinal cohort study included data from the Alzheimer’s Disease Neuroimaging Initiative from February 1, 2007, to June 6, 2016. Follow-up blood sampling was performed for up to 8 years. Plasma p-tau181 measurements were performed in 2020. This was a multicentric observational study of 1113 participants, including cognitively unimpaired participants as well as patients with cognitive impairment (mild cognitive impairment and AD dementia). Participants were eligible for inclusion if they had available plasma p-tau181 and NfL measurements and at least 1 fluorine-18–labeled fluorodeoxyglucose (FDG) positron emission tomography (PET) or structural magnetic resonance imaging scan performed at the same study visit. Exclusion criteria included any significant neurologic disorder other than suspected AD; presence of infection, infarction, or multiple lacunes as detected by magnetic resonance imaging; and any significant systemic condition that could lead to difficulty complying with the protocol.

**Exposures:**

Plasma p-tau181 and NfL measured with single-molecule array technology.

**Main Outcomes and Measures:**

Longitudinal imaging markers of neurodegeneration (FDG PET and structural magnetic resonance imaging) and cognitive test scores (Preclinical Alzheimer Cognitive Composite and Alzheimer Disease Assessment Scale–Cognitive Subscale with 13 tasks). Data were analyzed from June 20 to August 15, 2020.

**Results:**

Of the 1113 participants (mean [SD] age, 74.0 [7.6] years; 600 men [53.9%]; 992 non-Hispanic White participants [89.1%]), a total of 378 individuals (34.0%) were cognitively unimpaired (CU) and 735 participants (66.0%) were cognitively impaired (CImp). Of the CImp group, 537 (73.1%) had mild cognitive impairment, and 198 (26.9%) had AD dementia. Longitudinal changes of plasma p-tau181 were associated with cognitive decline (CU: *r* = –0.24, *P* < .001; CImp: *r* = 0.34, *P* < .001) and a prospective decrease in glucose metabolism (CU: *r* = –0.05, *P* = .48; CImp: *r* = –0.27, *P* < .001) and gray matter volume (CU: *r* = –0.19, *P* < .001; CImp: *r* = –0.31, *P* < .001) in highly AD-characteristic brain regions. These associations were restricted to amyloid-β–positive individuals. Both plasma p-tau181 and NfL were independently associated with cognition and neurodegeneration in brain regions typically affected in AD. However, NfL was also associated with neurodegeneration in brain regions exceeding this AD-typical spatial pattern in amyloid-β–negative participants. Mediation analyses found that approximately 25% to 45% of plasma p-tau181 outcomes on cognition measures were mediated by the neuroimaging-derived markers of neurodegeneration, suggesting links between plasma p-tau181 and cognition independent of these measures.

**Conclusions and Relevance:**

Study findings suggest that plasma p-tau181 was an accessible and scalable marker for predicting and monitoring neurodegeneration and cognitive decline and was, unlike plasma NfL, AD specific. The study findings suggest implications for the use of plasma biomarkers as measures to monitor AD progression in clinical practice and treatment trials.

## Key Points

**Question:**

What is the potential of blood-based biomarkers for predicting and monitoring the progression of Alzheimer disease neurodegeneration?

**Findings:**

In this cohort study that included 1113 participants from the multicentric Alzheimer’s Disease Neuroimaging Initiative study, baseline and longitudinal increases of tau phosphorylated at threonine 181 (p-tau181) in blood plasma were associated with progressive, longitudinal neurodegeneration in brain regions characteristic for Alzheimer disease, as well as with cognitive decline, only among participants with elevated brain amyloid-β. Neurofilament light chain in plasma, however, was associated with disease progression independent of amyloid-β and plasma p-tau181.

**Meaning:**

These findings suggest that plasma p-tau181, alone or combined with plasma neurofilament light chain, can be used as an accessible, minimally invasive biomarker to track Alzheimer disease progression.

## Teaser

This cohort study uses data from the Alzheimer’s Disease Neuroimaging Initiative study to investigate if blood-based biomarkers can be used to monitor the progression of Alzheimer disease neurodegeneration.

## Introduction

Alzheimer disease (AD) is a neurodegenerative disorder characterized by the accumulation of amyloid-β (Aβ) plaques and neurofibrillary tangles of hyperphosphorylated tau in the brain.^1^ These neuropathologic changes are believed to take part in a cascade of events that result in a characteristic neurodegeneration pattern followed by progressive cognitive impairment.^2^ Tracking neurodegenerative changes in vivo is important for monitoring AD progression. Current positron emission tomography (PET) and cerebrospinal fluid biomarkers enable the detection of Aβ and tau pathology,^3,4,5,6^ but the generalized use of these biomarkers is currently limited by their costs, availability, and invasiveness.

Recent evidence suggests that blood-based biomarkers might be useful to detect AD pathology,^7,8,9,10,11,12,13,14,15,16,17,18,19^ potentially promoting the widespread use of biomarkers in the diagnostic workup of AD and clinical trial screening. Among candidate disease-specific biomarkers in blood, plasma phosphorylated tau at threonine 181 (p-tau181) has shown promise as a marker of disease status.^7,9,10,11,12,19^ However, the potential of plasma p-tau181 as a marker of disease progression remains largely unexplored. Specifically, it remains unclear (1) how baseline and longitudinal plasma p-tau181 is associated with progressive AD-specific neurodegeneration; (2) whether plasma p-tau181 provides complementary information to non–disease-specific plasma biomarkers of neurodegeneration, such as neurofilament light chain (NfL)^20,21,22^; and (3) how imaging neurodegeneration markers mediate the association between plasma p-tau181 and cognitive decline.

In this study, we hypothesized that both baseline and longitudinal plasma p-tau181 levels associate with progressive AD-related neurodegeneration, which may mediate the associations between p-tau181 and cognitive decline. To test this hypothesis, we investigated longitudinal associations between plasma p-tau181 and established imaging markers of regional neurodegeneration on fluorine 18–labeled [^18^F]fluorodeoxyglucose (FDG) PET and structural magnetic resonance imaging (MRI), as well as relationships with cognitive performance, in more than 1000 individuals from the Alzheimer’s Disease Neuroimaging Initiative (ADNI). In addition, we explored whether plasma p-tau181 provides complementary information to plasma NfL in forecasting and tracking AD-related neurodegeneration and cognitive decline.

## Methods

### Study Design

Data used in this cohort study were obtained from the ADNI database^23^ from February 1, 2007, to June 6, 2016 (eMethods in Supplement 1). In this study, we included all cognitively unimpaired (CU) and cognitively impaired (CImp) participants, including those with mild cognitive impairment and AD dementia, from the ADNI Grand Opportunity/ADNI2 study with available plasma p-tau181 and NfL data and at least 1 FDG PET scan or structural T1 MRI performed at the same study visit (n = 1113). In addition, 1048 participants of the study sample (94%) also underwent PET imaging with the Aβ-sensitive tracer [^18^F]florbetapir. Demographic characteristics of study participants are presented in the Table. Further details of baseline and follow-up assessments are provided in the eMethods in Supplement 1. Inclusion criteria for the different diagnostic categories in the ADNI cohort have been described previously.^24^ All participants provided written informed consent approved by the institutional review board of each ADNI participating institution. This study followed the Strengthening the Reporting of Observational Studies in Epidemiology (STROBE) reporting guideline.

**Table.** Cohort Characteristics^a^

Characteristic	Cognitively unimpaired (n = 374)	Cognitively impaired (n = 734)
**Baseline characteristics**		
Age, mean (SD), y	74.8 (6.6)	73.6 (8)
Sex, men/women, No.	176/198	421/312
Race/ethnicity, non-Hispanic White, No. (%)	323 (86)	664 (90)
APOE ε4 carriers, No. (%+^b^)	108 (29)	376 (51)
MCI/AD	NA	536/198
Aβ-positive, No. (%)	113 (32)^c^	441 (65)^d^
Plasma p-tau181, median (range), pg/mL	13.3 (0.4 to 72.3)	18.4 (1.2 to 69.6)
Plasma NfL, median (range), pg/mL	33.3 (8.0 to 169.0)	37.9 (6.4 to 198.5)
Meta-ROI glucose metabolism, FDG PET SUVR, mean (SD)	1.57 (0.14)	1.47 (0.18)
AD-signature ROI volume, mean (SD), cm^3^	31.4 (3.2)	29.8 (4.2)
PACC, mean (SD)	0.0 (2.62)	NA
ADAS-Cog 13, mean (SD)	NA	19.0 (10.5)
**Follow-up characteristics**		
Plasma p-tau181		
Annual change, mean (SD), pg/mL/y	0.34 (0.39)	0.49 (0.37)
Median follow-up, y	2.1	3.0
Plasma NfL		
Annual change, mean (SD), pg/mL/y	1.9 (1.8)	2.6 (2.5)
Median follow-up, y	2.1	3.0
Meta-ROI glucose metabolism		
Annual change, mean (SD), SUVR/y	−0.016 (0.009)	−0.019 (0.014)
Median follow-up, y	2.0	2.0
AD-signature ROI volume		
Annual change, mean (SD), cm^3^/y	−0.12 (0.11)	−0.19 (0.14)
Median follow-up, y	5.0	2.1
PACC		
Annual change, mean (SD)	−0.20 (0.26)	NA
Median follow-up, y	6.0	NA
ADAS-Cog 13		
Annual change, mean (SD)	NA	1.9 (1.8)
Median follow-up, y	NA	4.0

Abbreviations: Aβ, amyloid-β; AD, Alzheimer disease; ADAS-Cog 13, Alzheimer Disease Assessment Scale–Cognitive Subscale with 13 tasks; APOE, apolipoprotein E; FDG, fluorine 18–labeled fluorodeoxyglucose; MCI, mild cognitive impairment; NA, not applicable; NfL, neurofilament light chain; PACC, Preclinical Alzheimer Cognitive Composite; p-tau181, phosphorylated tau at threonine 181; PET, positron emission tomography; ROI, region of interest; SUVR, standardized uptake value ratio.

^a^ The demographic characteristics of the outlier cases are not reported in this table.

^b^ The %+ indicates the proportion of individuals who carry the APOE ɛ4 allele.

^c^ Assessed in a subset of 348 participants.

^d^ Assessed in a subset of 695 participants.

### Blood Biomarkers

Blood sampling and processing were carried out in accordance with the ADNI protocol^25^ and analyzed at the Clinical Neurochemistry Laboratory, University of Gothenburg in Mölndal, Sweden. Plasma p-tau181 concentration was measured using a novel assay developed in-house on the single-molecule array HD-X (Simoa; Quanterix Corporation) instrument, as described previously.^11^ Plasma NfL concentration was also measured using Simoa, as previously described.^26^ All blood samples were analyzed in a single batch for each measure.^27^ We identified 4 outliers for plasma p-tau181 values and 1 for NfL (0.4%), which were excluded from subsequent analyses (eFigure 1 in Supplement 1).

### Neuroimaging

Acquisition protocols and preprocessing steps in ADNI for FDG PET and structural MRI are described in detail elsewhere^28,29^ and have been summarized in the eMethods in Supplement 1. Our in-house processing pipeline for FDG PET and structural MRI, as well as details of the methods for voxelwise and region-of-interest (ROI) analyses, are also detailed in the eMethods in Supplement 1. With FDG PET, we measured AD-typical glucose hypometabolism as the average standardized uptake value ratio (SUVR), using the pons as the reference region,^30^ in a previously defined Meta-ROI in Montreal Neurological Institute space^31^ that recapitulates regions of typical hypometabolism (angular gyrus, posterior cingulate, and inferior temporal gyrus) in AD. Structural T1-weighted MRI scans were used to measure gray matter volume of a previously defined AD-signature ROI composed of entorhinal, fusiform, inferior temporal, and middle temporal cortices.^32^ We also analyzed gray matter volume in a hippocampus ROI as another commonly used structural MRI measure of AD-related neurodegeneration^33^ (eTable in Supplement 1).

### Cognitive Assessments

In CU individuals, global cognitive performance was assessed using a cognitive composite measure specifically designed for detecting early cognitive changes in clinical trials involving CU individuals with evidence of AD pathology, the Preclinical Alzheimer Cognitive Composite (PACC),^34^ adapted for the available tests in ADNI.^35^ Lower PACC scores represent poorer cognitive performance. In CImp participants, the Alzheimer Disease Assessment Scale–Cognitive Subscale with 13 tasks (ADAS-Cog 13)^36^ was used to assess cognitive impairment severity. Higher ADAS-Cog 13 scores represent poorer cognitive performance.

### Statistical Analysis

Individual rates of change in plasma biomarker levels as well as in imaging measures (at the voxel and ROI levels) were estimated using linear mixed models with participant-specific intercepts and slopes predicting biomarker levels over time.

We investigated the associations between (1) baseline plasma biomarker levels and longitudinal change in hypometabolism, atrophy, and cognition and (2) longitudinal plasma biomarker changes and longitudinal hypometabolism, atrophy, and cognitive change. Analyses of the associations between baseline plasma biomarkers and baseline neurodegeneration are provided in the eAppendix and eFigures 2, 3, 4, 5, 6, and 7 in Supplement 1). For each analysis, the following steps were conducted: first, we fitted linear regressions separately for CU and CImp individuals, adjusted for age and sex (as well as field strength and total intracranial volume for atrophy measures) using voxel or ROI-level imaging-based neurodegeneration markers as the dependent variable and plasma p-tau181 and NfL, respectively, as the independent variable. Second, we studied the independent contributions of each plasma biomarker to hypometabolism or atrophy in the previously defined AD-specific ROIs. For this, we used both plasma p-tau181 and NfL as independent variables in linear models adjusted for the same covariates as described previously, and we compared the corresponding standardized β coefficients by computing 95% CIs derived using a 2000-repetition bootstrap procedure. Effect sizes were computed as partial correlation coefficients (*r*). These analyses were repeated substituting neurodegeneration markers as response variables by cognitive measures and adjusted for age, sex, and years of education. Additionally, we performed mediation analyses to investigate how imaging neurodegeneration markers influenced the association between plasma p-tau181 and cognition. Finally, we investigated how plasma biomarkers correlated with imaging-based neurodegeneration markers and cognition among participants stratified by cognitive status (CU or CImp) and Aβ status (positive, + or negative, −) according to a previously defined cut point of 1.11 [^18^F]florbetapir SUVR (using the whole cerebellum as reference region) for ADNI.^37^ All statistical analyses were conducted from June 20 to August 15, 2020, using MatLab 2018a (The MathWorks Inc). All tests were 2-sided. Significance level was set at *P* < .05. No corrections for multiple comparisons were carried out except for voxelwise analyses, following recommendations from the statistical literature that discourage the use of such procedures for hypothesis-driven studies with a limited number of planned comparisons.^38^

## Results

### Baseline Plasma P-Tau181 Predicts Longitudinal Neurodegeneration and Cognitive Decline

**Figure 1.**
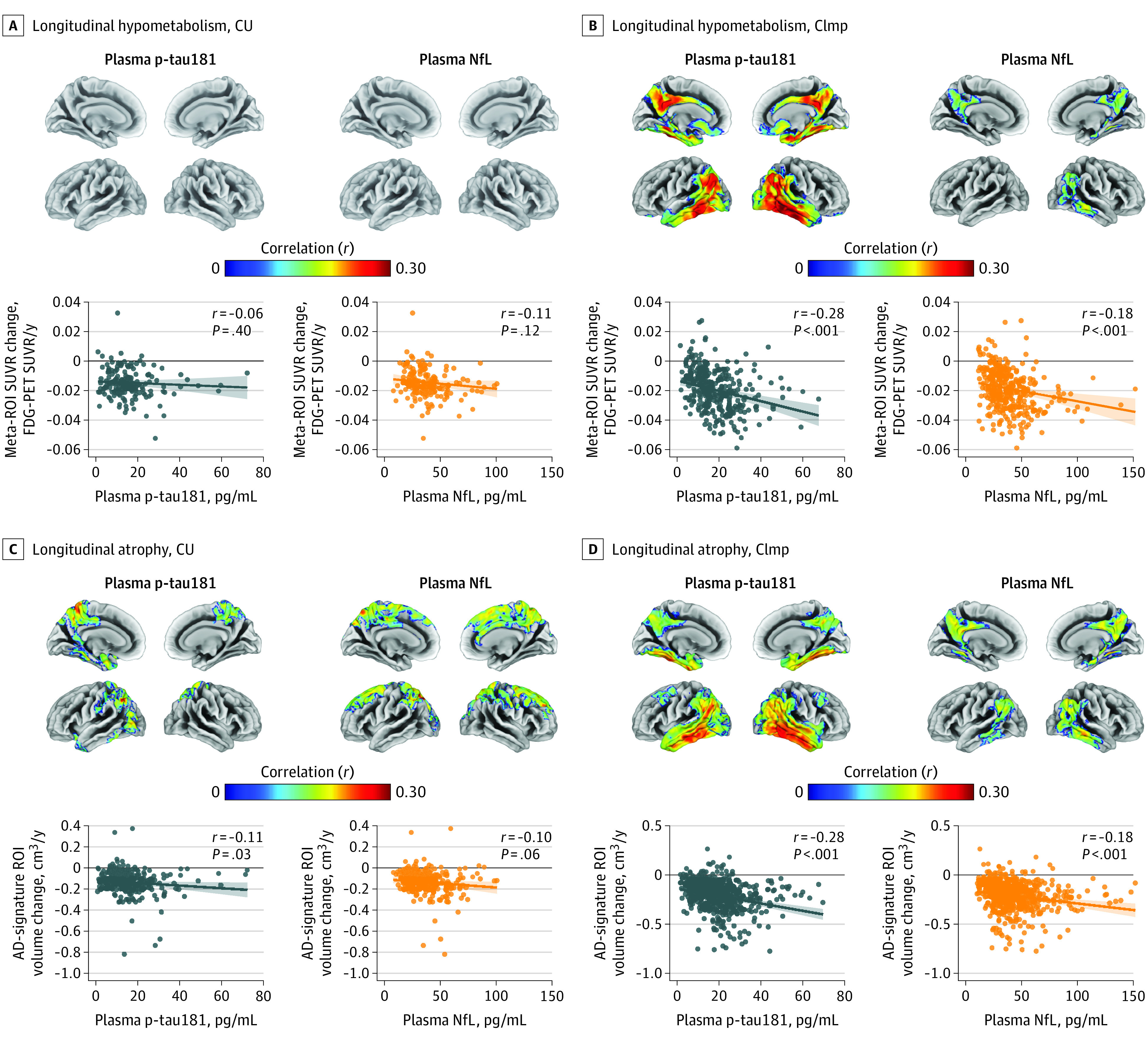
Associations of Baseline Plasma Phosphorylated Tau at Threonine 181 (P-Tau181) and Neurofilament Light Chain (NfL) With Decreasing Glucose Metabolism and Increasing Atrophy Regression lines displayed in graphs were computed by setting covariates in the linear model to average group levels (cognitively unimpaired [CU] or cognitively impaired [CImp]) and categorical variables to the reference (female sex and, for atrophy measures, 3-T field strength). Age- and sex-adjusted associations of baseline plasma p-tau181 and NfL with hypometabolism progression are shown at the voxel (upper row) and Alzheimer disease (AD) meta–region of interest (ROI) level (bottom row) in cognitively unimpaired (A) and cognitively impaired (B) individuals. To account for the difference in sample sizes, results of voxelwise analyses were thresholded on the voxel level at *P* < .01 (uncorrected) for the CU group and *P* < .001 (uncorrected) for CImp. All maps were further thresholded at the cluster level by restricting to clusters with a number of voxels higher than the expected number of voxels as predicted using random field theory. Age- and sex-adjusted associations of baseline plasma p-tau181 and NfL with atrophy progression are shown at the voxel (upper row) and AD-signature ROI level (bottom row) in cognitively unimpaired (C) and cognitively impaired (D) individuals. Results of voxelwise analyses were thresholded at *P* < .01 (uncorrected) for the CU group and at *P* < .001 (uncorrected) for CImp. All maps were further thresholded at *P* < .05 (familywise error corrected) at the cluster level. The eTable in Supplement 1 shows ROI analyses using hippocampus volume. FDG indicates fluorine 18–labeled fluorodeoxyglucose; PET, positron emission tomography; and SUVR, standardized uptake value ratio.

Of the 1113 participants (mean [SD] age, 74.0 [7.6] years; 600 men [53.9%]; and 992 non-Hispanic White participants [89.1%]), a total of 378 individuals (34.0%) were CU and 735 participants (66.0%) were CImp. Of the CImp group, 537 (73.1%) had mild cognitive impairment, and 198 (26.9%) had AD dementia. We first investigated how baseline plasma p-tau181 levels would predict future neurodegeneration progression. Higher plasma p-tau181 levels were associated with faster longitudinal progression of hypometabolism and atrophy among CImp individuals in AD-vulnerable areas (FDG PET SUVR change, *r* = –0.28, *P* < .001; gray matter volume change: *r* = –0.28, *P* < .001) (Figure 1A and B). eFigure 8 in Supplement 1 shows the typical spatial patterns of glucose hypometabolism and atrophy in AD. Moreover, plasma p-tau181 was associated with future atrophy in AD-vulnerable temporoparietal regions among CU individuals (*r* = –0.11, *P* = .03) (Figure 1C). This finding contrasts with the associations between plasma NfL and regional progressive atrophy observed in CU individuals, which were mainly pronounced in frontal regions and did not involve the temporal lobe (Figure 1C); eFigure 9 in Supplement 1 shows the spatial overlap between plasma p-tau181 and NfL association maps. None of the plasma biomarkers were significantly associated with decreasing glucose metabolism in the CU group; however, there was a reduced sample size with available longitudinal FDG PET scans (approximately 50% of total patients). Although plasma p-tau181 and NfL were positively associated (eFigure 10 in Supplement 1), both plasma biomarkers were independently associated with progressive AD-typical neurodegeneration with comparable effect sizes (eFigure 11 in Supplement 1); however, for atrophy progression in the CImp group, plasma p-tau181 had a statistically significantly stronger association than plasma NfL (_βp-tau181_ – β_NfL_ = –0.13; 95% CI, –0.27 to 0.00).

Baseline plasma p-tau181 levels were also associated with prospective cognitive decline, both in CU (*r* = −0.12, *P* = .04) and in CImp (*r* = 0.35, *P* < .001) individuals. In contrast, plasma NfL was only associated with cognitive decline among CImp individuals (CU: *r* = −0.06, *P* = .30; CImp: *r* = 0.26, *P* < .001). In combined models, both plasma markers were independently associated with prospective cognitive decline in CImp individuals (eFigure 12A in Supplement 1). Mediation analyses found that 25% to 45% of baseline plasma p-tau181 association with cognitive decline were mediated by baseline imaging neurodegeneration markers (eFigure 12B in Supplement 1).

**Figure 2.**
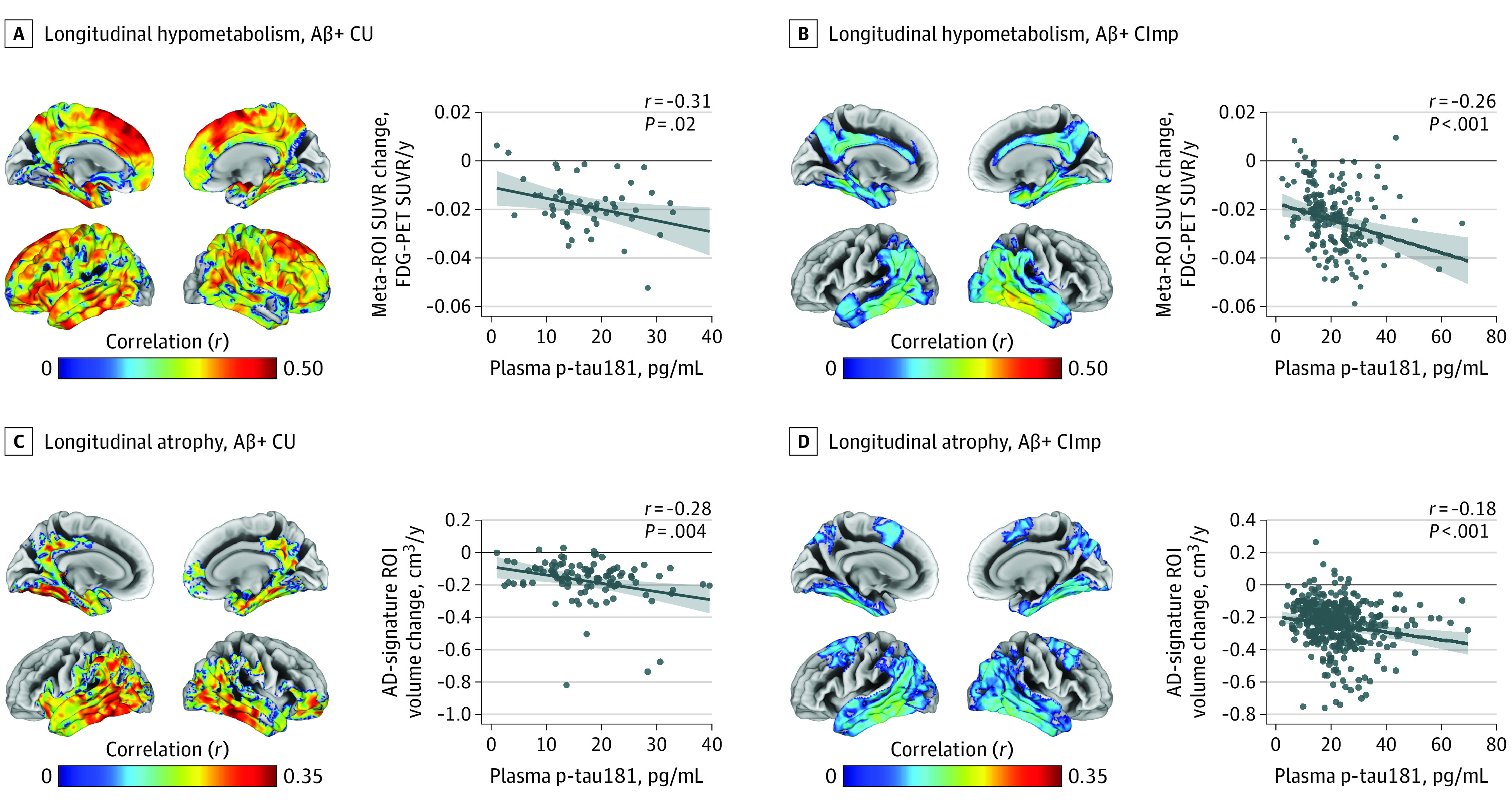
Associations of Baseline Plasma Phosphorylated Tau at Threonine 181 (P-Tau181) With Decreasing Glucose Metabolism and Increasing Atrophy in Amyloid-β–Positive (Aβ+) Cognitively Unimpaired and Impaired Participants Associations of baseline plasma p-tau181 with longitudinal hypometabolism in Aβ+ cognitively unimpaired (CU) (A) and Aβ+ cognitively impaired (CImp) (B) and with longitudinal atrophy in Aβ+ CU (C) and Aβ+ CImp (D) at the voxel and region-of-interest (ROI) level. Models were adjusted for age, sex, and, for atrophy measures, for total intracranial volume and MRI field strength. Statistical maps were thresholded using a lenient threshold (*P* < .05 [uncorrected] at the voxel level and further thresholded at the cluster level by restricting results to clusters with a number of voxels higher than the expected number of voxels as predicted using random field theory) to maximize detection power in the Aβ– group while keeping identical thresholds for the Aβ+ group. Reported partial correlation coefficients were adjusted for the same covariates. Regression lines were computed by setting covariates in the linear model to average group levels (CU or CImp) and categorical variables to the reference (female sex and, for atrophy measures, 3-T field strength). The eTable in Supplement 1 shows ROI analyses using hippocampus volume. FDG indicates fluorine 18–labeled fluorodeoxyglucose; SUVR, standardized uptake value ratio.

In Aβ-stratified analyses, plasma p-tau181 was only associated with hypometabolism and atrophy in AD-typical regions among Aβ+ CU and Aβ+ CImp participants (FDG PET SUVR change: Aβ+ CU, *r* = −0.31, *P* = .02; Aβ+ CImp, *r* = −0.26, *P* < .001; gray matter volume change: Aβ+ CU, *r* = −0.28, *P* = .004; and Aβ+ CImp, *r* = −0.18, *P* < .001) (Figure 2 and eFigure 13 in Supplement 1). Similarly, plasma p-tau181 was associated with cognitive decline in both Aβ+ CU and Aβ+ CImp participants (Aβ+ CU: *r* = −0.33, *P* < .001; Aβ+ CImp: *r* = 0.28, *P* < .001) but not in Aβ− individuals. In contrast, plasma NfL was associated with progressive atrophy in the Aβ− groups, mainly involving the dorsal frontal lobe regions less typically involved in AD (eFigure 14 in Supplement 1). Plasma NfL was also associated with a decrease in glucose metabolism and increase in atrophy in AD-vulnerable regions in Aβ+ participants (FDG PET SUVR change: Aβ+ CU, *r* = −0.24, *P* = .08; Aβ+ CImp, *r* = −0.23, *P* = .002; gray matter volume change: Aβ+ CU, *r* = −0.23, *P* = .02; Aβ+ CImp, *r* = −0.13, *P* = .01) (eFigure 15 in Supplement 1). In line with these results, plasma NfL was also associated with cognitive decline in Aβ− CImp (*r* = 0.23, *P* < .001) and Aβ+ CImp (*r* = 0.25, *P* < .001) participants, but not in any of the CU groups.

### Plasma P-Tau181 Changes Parallel Longitudinal Neurodegeneration and Cognitive Decline

**Figure 3.**
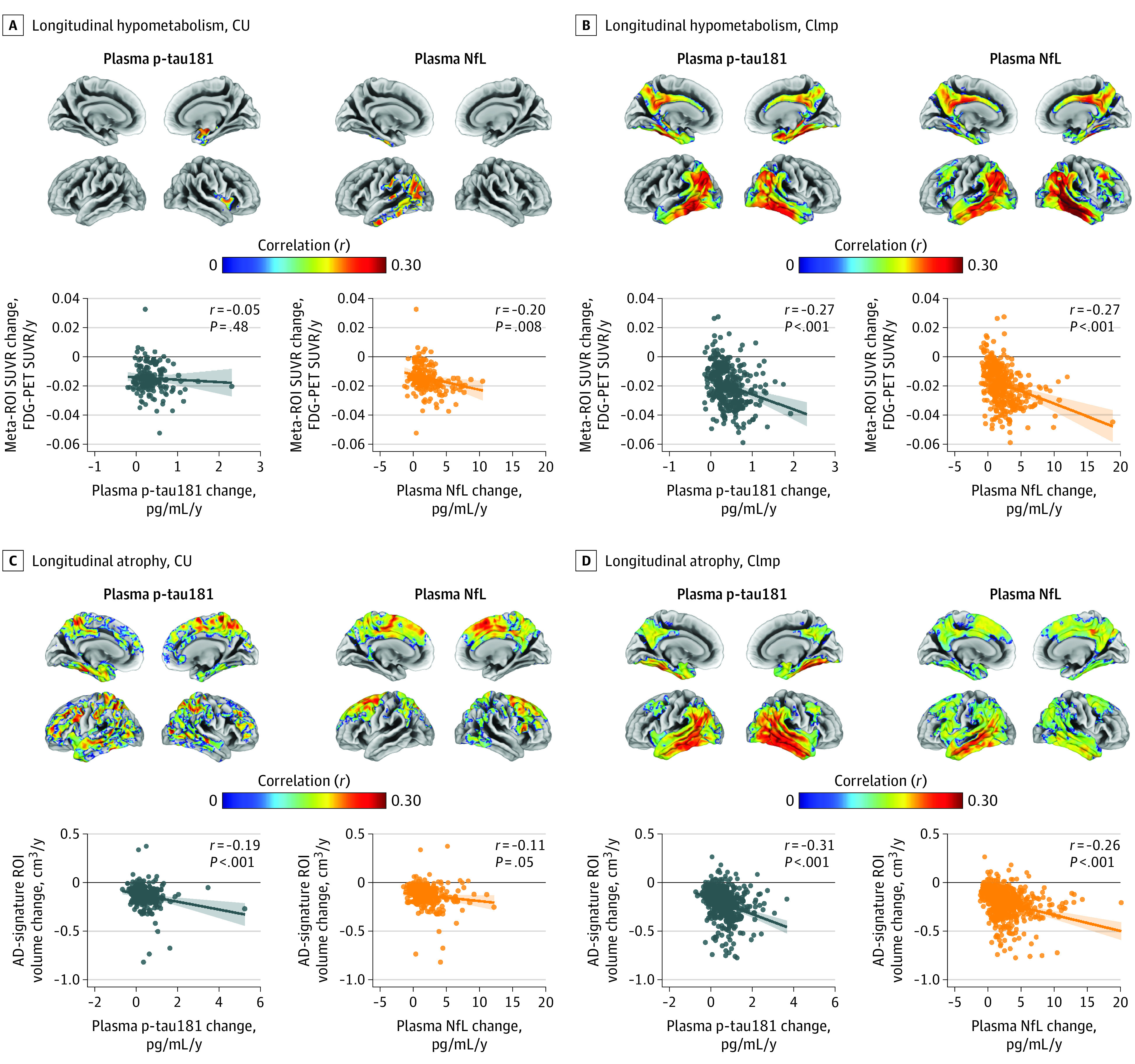
Associations of Plasma Phosphorylated Tau at Threonine 181 (P-Tau181) and Neurofilament Light Chain (NfL) Changes With Decreasing Glucose Metabolism and Increasing Atrophy Regression lines displayed in graphs were computed by setting covariates in the linear model to average group levels (cognitively unimpaired [CU] or cognitively impaired [CImp]) and categorical variables to the reference (female sex and, for atrophy measures, 3-T field strength). Age- and sex-adjusted associations of plasma p-tau181 and NfL change with hypometabolism progression are shown at the voxel (upper row) and Alzheimer disease (AD) meta–region of interest (ROI) level (bottom row) in CU (A) and CImp (B) individuals. To account for the difference in sample sizes, results of voxelwise analyses were thresholded at the voxel level at *P* < .01 (uncorrected) for the CU group and at *P* < .001 (uncorrected) for CImp. All maps were further thresholded at the cluster level by restricting results to clusters with a number of voxels higher than the expected number of voxels as predicted using random field theory. Age- and sex-adjusted associations of plasma p-tau181 and NfL changes with atrophy progression are shown at the voxel (upper row) and AD-signature ROI level (bottom row) in CU (C) and CImp (D) individuals. Results of voxelwise analyses were thresholded at *P* < .01 (uncorrected) for the CU group and at *P* < .001 (uncorrected) for CImp. All maps were further thresholded at *P* < .05 (familywise error corrected) at the cluster level. The eTable in Supplement 1 shows ROI analyses using hippocampus volume. FDG indicates fluorine 18–labeled fluorodeoxyglucose; SUVR, standardized uptake value ratio.

We then investigated whether longitudinal increases of plasma p-tau181 accompanied longitudinal neurodegeneration in AD-typical regions. Plasma p-tau181 changes were associated with a decrease in glucose metabolism and an increase in atrophy among CImp participants, although significant associations with progressive neurodegeneration were also found in CU individuals (FDG PET SUVR change: CImp, *r* = −0.27, *P* < .001; gray matter volume change: CU, *r* = −0.19, *P* < .001; CImp, *r* = −0.31, *P* < .001) (Figure 3), particularly with respect to atrophy progression. The spatial associations suggested again a high correspondence with AD-typical neurodegeneration patterns, although in the CU group, the pattern was more diffuse and also involved frontal areas. Plasma NfL changes were also significantly associated with progressive neurodegeneration in AD-typical areas (FDG PET SUVR change: CU, *r* = −0.20, *P* = .008; CImp, *r* = −0.27, *P* < .001; gray matter volume change: CU, *r* = −0.11, *P* = .05; CImp, *r* = −0.26, *P* < .001); however, the spatial pattern also involved other frontoparietal regions less characteristic of AD-typical neurodegeneration (Figure 3A and B). eFigure 16 in Supplement 1 shows the spatial overlap between plasma p-tau181 and NfL association maps. In multivariable analyses, changes of both plasma biomarkers were independently associated with progression of imaging-derived neurodegeneration markers (eFigure 17 in Supplement 1).

Similar to the associations with progressive neurodegeneration, longitudinal plasma p-tau181 changes were associated with prospective cognitive decline in both CU (*r* = −0.24, *P* < .001) and CImp (*r* = 0.34, *P* < .001) individuals. Plasma NfL changes were also associated with cognitive decline in CU (*r* = −0.12, *P* = .04) and CImp (*r* = 0.30, *P* < .001) individuals. However, in a combined model with plasma p-tau181, the association in CU individuals was no longer significant for NfL, whereas in CImp, longitudinal changes of both plasma markers were independently associated with variations in cognitive decline (CU: p-tau181, β = −0.23; 95% CI, −0.38 to −0.10; NfL, β = −0.04; 95% CI, −0.22 to 0.12; CImp: p-tau181, β = 0.28; 95% CI, 0.17-0.39; NfL, β = 0.23; 95% CI, 0.10-0.38) (eFigure 18A in Supplement 1). Mediation analyses found that 25% to 45% of the plasma p-tau181 association with longitudinal cognition was mediated by changes in imaging-derived neurodegeneration markers (eFigure 18B in Supplement 1).

**Figure 4.**
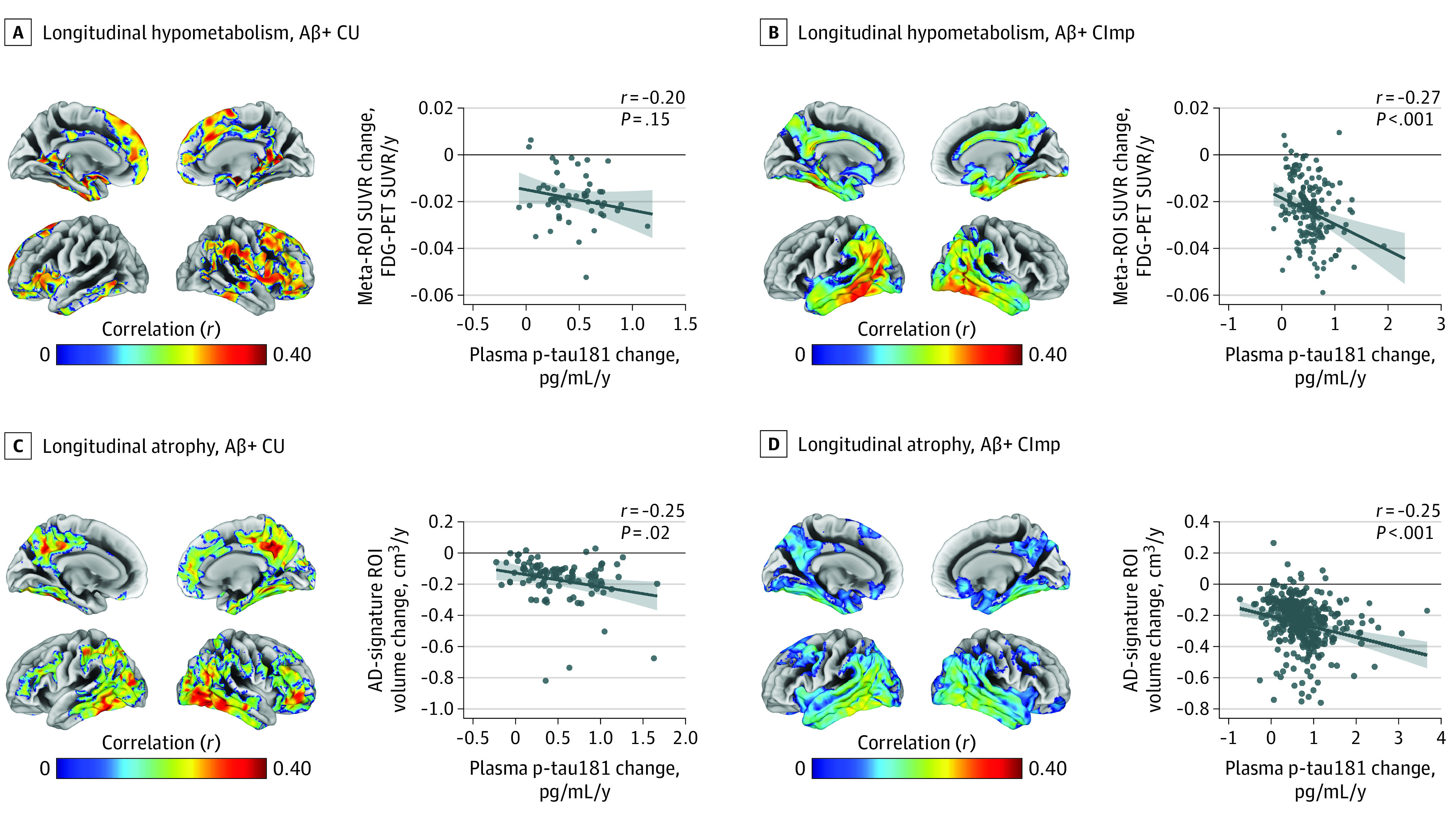
Associations of Longitudinal Plasma Phosphorylated Tau at Threonine 181 (P-Tau181) Change With Decreasing Glucose Metabolism and Increasing Atrophy in Amyloid-β–Positive (Aβ+) Cognitively Unimpaired (CU) and Cognitively Impaired (CImp) Participants Associations of longitudinal plasma p-tau181 change with longitudinal hypometabolism in Aβ+ CU (A) and Aβ+ CImp (B) and with longitudinal atrophy in Aβ+ CU (C) and Aβ+ CImp (D) at the voxel and region-of-interest (ROI) levels. Linear models were adjusted for age, sex, and, for atrophy measures, total intracranial volume and MRI field strength. Statistical maps were thresholded using a lenient threshold (*P* < .05 [uncorrected] at the voxel level and further thresholded at the cluster level by restricting to clusters with a number of voxels higher than the expected number of voxels as predicted using random field theory) to maximize detection power in the Aβ- group while keeping identical thresholds for the Aβ+ group. Reported partial correlation coefficients were adjusted for the same covariates. Regression lines were computed by setting covariates in the linear model to average group levels (CU or CImp) and categorical variables to the reference (female sex and, for atrophy measures, 3-T field strength). The eTable in Supplement 1 shows ROI analyses using hippocampus volume. FDG indicates fluorine 18–labeled fluorodeoxyglucose; SUVR, standardized uptake value ratio.

The results of analyses stratified by Aβ status suggest that plasma p-tau181 changed in parallel with neurodegeneration progression only among Aβ+ participants and in a spatial pattern that closely corresponds to AD-typical regional neurodegeneration, as evidenced by both voxelwise and ROI analyses (FDG PET SUVR change: Aβ+ CImp, *r* = −0.27, *P* < .001; gray matter volume change: Aβ+ CU, *r* = −0.25, *P* = .02; Aβ+ CImp, *r* = −0.25, *P* < .001) (Figure 4; eFigure 19 in Supplement 1). Similarly, plasma p-tau181 changes accompanied cognitive decline in Aβ+ participants (Aβ+ CU: *r* = −0.30, *P* = .003; Aβ+ CImp: *r* = 0.31, *P* < .001) but not in Aβ− participants (Aβ− CU: *r* = −0.14, *P* = .05; Aβ− CImp: *r* = −0.01, *P* = .92). By contrast, plasma NfL changes paralleled neurodegenerative changes also in Aβ− individuals, particularly with respect to progressive atrophy across widespread cortical areas that also covered large parts of the frontal lobe (eFigure 20 in Supplement 1). In ROI analyses, plasma NfL changes were associated with atrophy progression in AD-vulnerable ROIs for both Aβ groups, but associations with hypometabolism progression were only significant for Aβ+ participants (gray matter volume change: Aβ− CImp, *r* = −0.29, *P* < .001; Aβ+ CU, *r* = −0.25, *P* = .01; Aβ+ CImp, *r* = −0.17, *P* = .002; FDG PET SUVR change: Aβ+ CU, *r* = −0.42, *P* = .002; and Aβ+ CImp, *r* = −0.30, *P* < .001) (eFigures 20 and 21 in Supplement 1). Similar nonspecific results were observed for cognitive changes: plasma NfL changes were associated with a cognitive decline in CImp Aβ− and Aβ+ participants (Aβ− CImp: *r* = 0.25, *P* < .001; Aβ+ CImp: *r* = 0.26, *P* < .001) but not in CU Aβ− (*r* = −0.13, *P* = .06) or CU Aβ+ (*r* = −0.11, *P* = .30) participants.

## Discussion

In this cohort study, we investigated longitudinal associations of p-tau181 levels in blood with multimodal imaging biomarkers of regional neurodegeneration and cognition in 1113 ADNI participants covering the entire AD spectrum. Furthermore, we compared this novel AD biomarker with a blood-based biomarker of neuronal injury, plasma NfL, which is increased in several neurodegenerative disorders and thus not considered specific for AD.^22^ Our findings suggest that (1) baseline plasma p-tau181 levels were associated with cognitive decline as well as with concurrent and prospective neurodegeneration in areas typically vulnerable in AD, as measured by structural MRI and FDG PET; (2) longitudinal increments of plasma p-tau181 accompanied cognitive decline and longitudinal progression of neurodegeneration in the same AD-vulnerable regions; (3) plasma p-tau181 and NfL were independently associated with cognition and neurodegeneration in AD-vulnerable areas; (4) plasma p-tau181 was specifically associated with cognitive impairment and an AD-typical regional neurodegeneration pattern among participants in the AD continuum (Aβ+), whereas NfL was associated with cognitive decline and neurodegeneration in both Aβ+ and Aβ− groups, generally in spatial neurodegeneration patterns that were less specific for AD-vulnerable regions; and (5) the associations between plasma p-tau181 and cognition were not fully mediated by imaging-derived neurodegeneration markers, suggesting independent links between plasma p-tau181 and cognitive impairment that are not explained by neurodegeneration as assessed with neuroimaging. Taken together, these results suggest the potential of plasma p-tau181 as a scalable, cost-effective, and accessible tool for estimating and monitoring AD-specific disease progression, extending results from previous studies that mainly focused on the ability of plasma p-tau181 for establishing disease status.^7,9,10,11,12^

A main finding of the present study was the observation that longitudinal increments of plasma p-tau181 paralleled worsening hypometabolism, atrophy, and cognitive decline. These associations, although generally stronger in the CImp group, were also significant in CU individuals, which suggests that plasma p-tau181 elevations might capture AD-related neurodegenerative processes even at early, presymptomatic disease stages and supports the use of repeated measurements of plasma p-tau181 biomarker levels over time for disease monitoring. However, some of the observed effect sizes were relatively small, particularly in the CU group. Thus, future studies are warranted to elucidate the clinical relevance of longitudinal plasma biomarkers for disease monitoring in different at-risk populations.

Our longitudinal findings resonate with recent results on longitudinal measures of plasma p-tau217, another novel candidate plasma biomarker of AD.^39^ Although the associations of longitudinal plasma p-tau217 with progressive neurodegeneration and cognitive decline were largely consistent with those observed here for p-tau181, plasma p-tau217 changes were not associated with progressive hippocampal atrophy in Aβ+ CImp participants.^39^ This finding contrasts with our results on plasma p-tau181, in which we observed statistically significant and generally stronger outcomes among CImp individuals. This discrepancy highlights the need for head-to-head comparison studies investigating the value of the respective novel blood-based biomarker for monitoring disease progression.

Consistent with prior findings assessing neurodegeneration with structural MRI,^7,11^ our results from FDG PET and structural MRI evaluation suggest that higher baseline plasma p-tau181 levels were associated with current and future neurodegeneration among CImp individuals. Baseline p-tau181 levels were weakly associated with prospective neurodegeneration in the CU group; however, we did observe more pronounced associations in the Aβ+ CU group, which suggests the predictive value of plasma p-tau181 when Aβ status information is available.

Using brainwide analyses at the voxel level, we found that plasma p-tau181 elevations were primarily associated with hypometabolism and atrophy in specific temporoparietal brain regions that are characteristically involved in AD-related neurodegeneration,^31,40,41,42^ and these associations were only present among Aβ+ individuals. Together, these findings suggest that p-tau181 is a specific marker for AD-related neurodegeneration. By contrast, plasma NfL was also significantly associated with hypometabolism and atrophy among Aβ− individuals, and these associations commonly covered larger frontoparietal areas not typically involved in AD. Neurodegeneration in frontoparietal areas has previously been found to be associated with white matter hyperintensities in aging,^43,44,45^ suggesting that the observed plasma NfL neurodegeneration patterns could be reflective of small vessel disease–related neuronal injury. Accordingly, plasma NfL levels have also been found to increase with increasing white matter hyperintensity burden.^46^ This finding is in line with findings from several previous studies indicating that plasma NfL is a more general marker of neuronal degeneration that is not specific for AD^26,47,48^. Interestingly, in combined regression models, we found that both plasma markers were independently associated with neurodegeneration in AD-typical areas, which suggests that both provide unique information about the underlying neurodegenerative processes that occur during the natural course of AD. This finding also suggests the potential for the combined use of these biomarkers for an optimized assessment of progressive neurodegeneration.

In line with findings from previous studies,^7,11,12^ we observed that baseline plasma p-tau181 levels were associated with prospective cognitive decline. Here, we extended this previous knowledge by noting that longitudinal increases of plasma p-tau181 paralleled cognitive decline even in asymptomatic stages of AD, further supporting the notion that plasma p-tau181 might capture early pathologic changes in the AD cascade. Moreover, we also found that approximately 50% to 70% of the associations of plasma p-tau181 with cognition were not mediated by hypometabolism or atrophy, suggesting that plasma p-tau181 reflects pathologic processes that influence cognitive performance through partly independent pathways not captured by these established imaging markers of neurodegeneration. This finding likely corresponds to the accumulation of neurofibrillary tangle pathology, which has been previously found to independently contribute to cognitive impairment beyond hypometabolism and atrophy measures.^49,50,51^ However, in the current study, we could not confirm this hypothesis owing to the lack of concurrent tau PET scans and plasma p-tau181 measures in the ADNI cohort. Further studies are needed to elucidate how tau PET mediates the associations between plasma p-tau181 and cognition.

Together, these findings further support the use of plasma p-tau181 not only for determining disease status but also as a cost-effective and specific biomarker of disease progression in AD. Plasma p-tau181, alone or in combination with plasma NfL, might represent a suitable tool for assessing and monitoring AD progression in clinical settings before conducting more expensive or invasive confirmatory imaging or cerebrospinal fluid tests. Owing to their close association with AD-typical neurodegeneration and cognition, repeated plasma p-tau181 measurements over time might also be useful to identify rapidly progressing forms of the disease in clinical scenarios as well as to track treatment outcomes in disease-modifying trials. Further studies in real clinical settings are warranted to investigate how the use of plasma biomarkers may affect clinically relevant outcomes.^52,53^

### Strengths and Limitations

This study features several strengths and limitations. First, we used a large, prospective cohort with longitudinal plasma biomarker data, as well as measures of cognition and multimodal imaging markers of neurodegeneration over a relatively long follow-up time. Second, almost all participants in the study also underwent Aβ PET, which allowed us to confirm that plasma p-tau181 elevations specifically correlated with neurodegeneration in participants along the AD continuum. Third, all the participants also had plasma NfL measurements, allowing a head-to-head comparison of the neurodegenerative features associated with each of the plasma-derived biomarkers. The study has several principal limitations. First, the study used a single cohort derived from ADNI, which represents a rather selective population. Because the measurement of plasma p-tau181 has only recently been introduced, there currently exists, to our knowledge, no other comparably large cohort that could provide access to blood-derived measures of p-tau181 and NfL in combination with the detailed neuroimaging information from structural MRI, FDG PET, and Aβ PET that was analyzed in our study, thus limiting the possibility to replicate our findings in an independent cohort at this time. Still, the large study sample as well as the robustness of the results, with converging findings from 2 different imaging modalities for measuring neurodegeneration along with measures of cognitive decline, provide strong evidence in support of the potential of plasma p-tau181 for disease monitoring in AD. Second, only approximately 50% of the study participants had longitudinal FDG PET scans, which limited the statistical power to detect associations with a decline in glucose metabolism, particularly in the CU group. Third, study participants did not have available tau PET scans at the moment of plasma p-tau181 measurement. Fourth, the ADNI study recruits participants who are relatively devoid of vascular pathology. Recent evidence suggests that white matter damage associates with Aβ deposition,^54,55,56^ and therefore, given the strong dependence of plasma p-tau181 on Aβ, it is unclear how vascular pathology might affect our findings.

## Conclusions

In conclusion, our findings suggest that both baseline levels and longitudinal changes in plasma p-tau181 levels were associated with prospective neurodegeneration and cognitive decline that can be described as characteristic for AD. Plasma NfL showed similarly pronounced associations with cognition and imaging markers of neurodegeneration, but, in contrast to plasma p-tau181, these associations were not AD specific. These findings support the combined use of plasma p-tau181 and NfL for improved prediction and monitoring of disease progression in AD.

## Notes

Supplement 1. **eMethods.** Alzheimer’s Disease Neuroimaging Initiative (ADNI) **eAppendix.** Baseline Plasma P-Tau181 Associates With Baseline Neurodegeneration and Cognition **eFigure 1.** Plasma P-Tau181 and Plasma NfL Distributions **eFigure 2.** Associations of Baseline Plasma P-Tau181 and NfL With Baseline Hypometabolism and Atrophy **eFigure 3.** Associations of Baseline Plasma Biomarker Levels With Baseline Cognition **eFigure 4.** Associations of Baseline Plasma P-Tau181 With Baseline Neurodegeneration Markers in Aβ+ Cognitively Unimpaired and Impaired Participants **eFigure 5.** Associations of Baseline Plasma P-Tau181 With Baseline Neurodegeneration Markers in Aβ- Cognitively Unimpaired and Impaired Participants **eFigure 6.** Associations of Baseline Plasma NfL With Baseline Neurodegeneration Markers in Aβ- Cognitively Unimpaired and Impaired Participants **eFigure 7.** Associations of Baseline Plasma NfL With Baseline Neurodegeneration Markers in Aβ+ Cognitively Unimpaired and Impaired Participants **eFigure 8.** Typical Spatial Patterns of Hypometabolism and Atrophy in AD **eFigure 9.** Brain Regions in Which Baseline Plasma P-Tau181 and Plasma NfL Correlate With Longitudinal Decrease in Glucose Metabolism and Greater Gray Matter Atrophy **eFigure 10.** Associations Between Plasma P-Tau181 and Plasma NfL **eFigure 11.** Independent Effects of Baseline Plasma Biomarkers on Longitudinal Neurodegeneration **eFigure 12.** Associations of Baseline Plasma Biomarker Levels With Cognitive Decline **eFigure 13.** Associations of Baseline Plasma P-Tau181 With Longitudinal Neurodegeneration Markers in Aβ- Cognitively Unimpaired and Impaired Participants **eFigure 14.** Associations of Baseline Plasma NfL With Longitudinal Neurodegeneration Markers in Aβ- Cognitively Unimpaired and Impaired Participants **eFigure 15.** Associations of Baseline Plasma NfL With Longitudinal Neurodegeneration Markers in Aβ+ Cognitively Unimpaired and Impaired Participants **eFigure 16.** Brain Regions in Which Longitudinal Plasma P-Tau181 and Plasma NfL Correlate With Longitudinal Decrease in Glucose Metabolism and Greater Gray Matter Atrophy **eFigure 17.** Independent Effects of Plasma Biomarker Change on Longitudinal Neurodegeneration **eFigure 18.** Associations of Plasma Biomarker Longitudinal Change With Cognitive Decline **eFigure 19.** Associations of Plasma P-Tau181 Longitudinal Change With Longitudinal Neurodegeneration Markers in Aβ- Cognitively Unimpaired and Impaired Participants **eFigure 20.** Associations of Plasma NfL Longitudinal Change With Longitudinal Neurodegeneration Markers in Aβ- Cognitively Unimpaired and Impaired Participants **eFigure 21.** Associations of Plasma NfL Longitudinal Change With Longitudinal Neurodegeneration Markers and Spatial Overlap With Plasma P-Tau181 in Aβ+ Cognitively Unimpaired and Impaired Participants **eTable.** Associations of Plasma P-Tau181 and Plasma NfL With Hippocampus Volumes **eReferences**

Supplement 2. **Nonauthor Collaborators**
