# Supplementary material for: Longitudinal Associations of Blood Phosphorylated Tau181 and Neurofilament Light Chain With Neurodegeneration in Alzheimer Disease
Source: JAMA Neurol. 2021 Jan 11;78(4):1–12. doi: 10.1001/jamaneurol.2020.4986 (PMC7802009; doi:10.1001/jamaneurol.2020.4986)
Supplement: Supplement 1. — eMethods. Alzheimer’s Disease Neuroimaging Initiative (ADNI) eAppendix. Baseline Plasma P-Tau181 Associates With Baseline Neurodegeneration and Cognition eFigure 1. Plasma P-Tau181 and Plasma NfL Distributions eFigure 2. Associations of Baseline Plasma P-Tau181 and NfL With Baseline Hypometabolism and Atrophy eFigure 3. Associations of Baseline Plasma Biomarker Levels With Baseline Cognition eFigure 4. Associations of Baseline Plasma P-Tau181 With Baseline Neurodegeneration Markers in Aβ+ Cognitively Unimpaired and Impaired Participants eFigure 5. Associations of Baseline Plasma P-Tau181 With Baseline Neurodegeneration Markers in Aβ- Cognitively Unimpaired and Impaired Participants eFigure 6. Associations of Baseline Plasma NfL With Baseline Neurodegeneration Markers in Aβ- Cognitively Unimpaired and Impaired Participants eFigure 7. Associations of Baseline Plasma NfL With Baseline Neurodegeneration Markers in Aβ+ Cognitively Unimpaired and Impaired Participants eFigure 8. Typical Spatial Patterns of Hypometabolism and Atrophy in AD eFigure 9. Brain Regions in Which Baseline Plasma P-Tau181 and Plasma NfL Correlate With Longitudinal Decrease in Glucose Metabolism and Greater Gray Matter Atrophy eFigure 10. Associations Between Plasma P-Tau181 and Plasma NfL eFigure 11. Independent Effects of Baseline Plasma Biomarkers on Longitudinal Neurodegeneration eFigure 12. Associations of Baseline Plasma Biomarker Levels With Cognitive Decline eFigure 13. Associations of Baseline Plasma P-Tau181 With Longitudinal Neurodegeneration Markers in Aβ- Cognitively Unimpaired and Impaired Participants eFigure 14. Associations of Baseline Plasma NfL With Longitudinal Neurodegeneration Markers in Aβ- Cognitively Unimpaired and Impaired Participants eFigure 15. Associations of Baseline Plasma NfL With Longitudinal Neurodegeneration Markers in Aβ+ Cognitively Unimpaired and Impaired Participants eFigure 16. Brain Regions in Which Longitudinal Plasma P-Tau181 and Plasma NfL Correlate With [file jamaneurol-e204986-s001.pdf]

Supplementary Online Content

Moscoso A, Grothe MJ, Ashton NJ, et al; Alzheimer's Disease Neuroimaging Initiative. Longitudinal associations of blood phosphorylated tau181 and neurofilament light chain with neurodegeneration in Alzheimer disease. *JAMA Neurol*. Published online January 11, 2021. doi:10.1001/jamaneurol.2020.4986

eMethods. Alzheimer's Disease Neuroimaging Initiative (ADNI)

eAppendix. Baseline Plasma P-Tau181 Associates With Baseline Neurodegeneration and Cognition

eFigure 1. Plasma P-Tau181 and Plasma NfL Distributions

eFigure 2. Associations of Baseline Plasma P-Tau181 and NfL With Baseline Hypometabolism and Atrophy

eFigure 3. Associations of Baseline Plasma Biomarker Levels With Baseline Cognition

eFigure 4. Associations of Baseline Plasma P-Tau181 With Baseline Neurodegeneration Markers in Aβ+ Cognitively Unimpaired and Impaired Subjects

eFigure 5. Associations of Baseline Plasma P-Tau181 With Baseline Neurodegeneration Markers in Aβ- Cognitively Unimpaired and Impaired Subjects

eFigure 6. Associations of Baseline Plasma NfL With Baseline Neurodegeneration Markers in Aβ- Cognitively Unimpaired and Impaired Subjects

eFigure 7. Associations of Baseline Plasma NfL With Baseline Neurodegeneration Markers in Aβ+ Cognitively Unimpaired and Impaired Subjects

eFigure 8. Typical Spatial Patterns of Hypometabolism and Atrophy in AD

eFigure 9. Brain Regions in Which Baseline Plasma P-Tau181 and Plasma NfL Correlate With Longitudinal Decrease in Glucose Metabolism and Greater Gray Matter Atrophy

eFigure 10. Associations Between Plasma P-Tau181 and Plasma NfL

eFigure 11. Independent Effects of Baseline Plasma Biomarkers on Longitudinal Neurodegeneration

eFigure 12. Associations of Baseline Plasma Biomarker Levels With Cognitive Decline

eFigure 13. Associations of Baseline Plasma P-Tau181 With Longitudinal Neurodegeneration Markers in Aβ- Cognitively Unimpaired and Impaired Subjects

eFigure 14. Associations of Baseline Plasma NfL With Longitudinal Neurodegeneration Markers in Aβ- Cognitively Unimpaired and Impaired Subjects

© 2021 Moscoso A et al. *JAMA Neurology.*

eFigure 15. Associations of Baseline Plasma NfL With Longitudinal Neurodegeneration Markers in Aβ+ Cognitively Unimpaired and Impaired Subjects

eFigure 16. Brain Regions in Which Longitudinal Plasma P-Tau181 and Plasma NfL Correlate With Longitudinal Decrease in Glucose Metabolism and Greater Gray Matter Atrophy

eFigure 17. Independent Effects of Plasma Biomarker Change on Longitudinal Neurodegeneration

eFigure 18. Associations of Plasma Biomarker Longitudinal Change With Cognitive Decline

eFigure 19. Associations of Plasma P-Tau181 Longitudinal Change With Longitudinal Neurodegeneration Markers in Aβ- Cognitively Unimpaired and Impaired Subjects

eFigure 20. Associations of Plasma NfL Longitudinal Change With Longitudinal Neurodegeneration Markers in Aβ- Cognitively Unimpaired and Impaired Subjects

eFigure 21. Associations of Plasma NfL Longitudinal Change With Longitudinal Neurodegeneration Markers and Spatial Overlap With Plasma P-Tau181 in Aβ+ Cognitively Unimpaired and Impaired Subjects

eTable. Associations of Plasma P-Tau181 and Plasma NfL With Hippocampus Volumes

eReferences

This supplementary material has been provided by the authors to give readers additional information about their work.

© 2021 Moscoso A et al. *JAMA Neurology.*

eMethods. Alzheimer's Disease Neuroimaging Initiative (ADNI)

Data used in the preparation of this article were obtained from the Alzheimer's Disease Neuroimaging Initiative (ADNI) database (adni.loni.usc.edu). The ADNI was launched in 2003 as a public-private partnership, led by Principal Investigator Michael W. Weiner, MD. The primary goal of ADNI has been to test whether serial magnetic resonance imaging (MRI), positron emission tomography (PET), other biological markers, and clinical and neuropsychological assessment can be combined to measure the progression of mild cognitive impairment (MCI) and early Alzheimer's disease (AD). For up-to-date information, see www.adni-info.org.

Baseline and follow-up assessments

The baseline visit was defined as the first study visit for which a plasma sample and an FDG PET or structural T1 MRI scan were available. Longitudinal blood sampling was performed annually in 984 participants over a median follow-up of 2.9 years. FDG PET was available at baseline for 1089 participants (average time lag between FDG PET imaging and plasma sampling = 11 days). Study participants were scheduled to undergo follow-up FDG PET scans at the same study site and scanner. Longitudinal FDG-PET scans were available for 513 participants over a median follow-up time of 2.0 years. Structural T1 MRI scans were performed at baseline in 1068 subjects (147 at 1.5T and 921 at 3T, average time lag between MRI and plasma sampling = 25 days). Study participants were scheduled to undergo follow-up MRI scans at the same study site and scanner. Longitudinal T1 scans were acquired three, six, and 12 months from baseline, and on subsequent annual study occasions in 981 subjects over a median follow-up time of 3.2 years. PACC was available at baseline for 343 CU individuals (average time lag between PACC testing and plasma sampling = 6 days); longitudinal PACC testing was performed annually in 320 participants over a median follow-up of six years. ADAS-Cog 13 was performed at baseline in 656 CI participants (average time lag between ADAS-Cog 13 examination and plasma sampling = 6 days); longitudinal ADAS-Cog 13 testing was performed annually in 613 subjects over a median follow-up of four years.

FDG PET and structural T1 preprocessing pipeline

For FDG PET imaging, a 30-minute six frame scan was acquired 30-60 minutes after the injection of 185 MBq of FDG. Acquired frames were then realigned, averaged, reoriented, resliced to a common grid, and smoothed to a common resolution of 8 mm. Further details on FDG PET acquisition and preprocessing in ADNI can be found at http://adni.loni.usc.edu/methods/pet-analysis-method/pet-analysis/ .

Structural T1-weighted MR images were acquired on 1.5T or 3T scanners using MP-RAGE/IR-SPGR sequences as described previously. T1 preprocessing steps in ADNI included scanner-specific correction for distortions due to gradient nonlinearity (Gradwarp), correction for image intensity non-uniformity (B1), and bias field correction (N3). Further details can be found at http://adni.loni.usc.edu/methods/documents/mri-protocols/.

FDG PET processing pipeline

Pre-processed FDG PET images were non-linearly registered to an FDG PET template in MNI space using the *"Old Normalise"* tool in SPM12. Spatially normalized images were used to derive standardized uptake value ratio (SUVR) maps by voxel-wise scaling to the average signal in a pons region-of-interest (ROI)[1]. For voxel-wise analyses, FDG SUVR maps in MNI space were masked using a cerebral GM mask and smoothed using an isotropic 4 mm filter. We applied this filter resolution in order to obtain more comparable resolutions across FDG PET and structural MRI. Longitudinal FDG PET scans were pre-processed identically to the baseline scans.

© 2021 Moscoso A et al. *JAMA Neurology.*

Structural MRI processing pipeline

For cross-sectional analyses, baseline T1 scans were segmented into gray (GM) matter, white (WM) matter, and CSF probability maps, and spatially normalized (with modulation) to MNI space using the Computational Anatomy Toolbox (CAT12, http://dbm.neuro.uni-jena.de/cat/) in Statistical Parametric Mapping 12 (SPM12; Wellcome Department of Imaging Neuroscience, Institute of Neurology, London, UK).

For longitudinal analyses of GM changes over time, first, we used the Serial Longitudinal Registration tool in SPM12 for intra-subject spatial normalization of baseline and follow-up T1 scans[2]. Briefly, this pipeline uses both rigid and non-linear registration algorithms to create a subject-specific template across time points as well as Jacobian determinant maps (in native space) for each T1 scan describing the relative volumetric differences between this template and each individual scan. Then, the standard CAT12 pipeline was used to segment the subject-specific templates and to spatially normalize the respective GM probability maps in MNI space, modulating voxel values to preserve the amount of volume present before normalization. The same deformation field was used to warp the Jacobian maps from subject-specific longitudinal registration into MNI space. Finally, Jacobian maps in MNI space were multiplied by the corresponding modulated GM probability map of the subject-specific template image. These *Jacobian×GM* maps therefore represent regional GM volumes at each time point in MNI space.

The previously derived GM (for cross-sectional analyses) and *Jacobian×GM* (for longitudinal analyses) maps in MNI space were used for the region-of-interest (ROI) as well as the voxel-wise analyses. For analyses at the ROI level, we calculated GM volumes of a previously defined AD-signature ROI composed of entorhinal, fusiform, inferior temporal, and middle temporal cortices (average left and right) by summing up the modulated GM values within this ROI, as defined using the Neuromorphometrics atlas (Neuromorphometrics, Inc.). In voxel-wise analyses, modulated GM and *Jacobian×GM* maps in MNI space were masked using a cerebral GM mask and smoothed using an isotropic 6 mm filter prior to statistical analyses. Total intracranial volumes (TIV) were computed as part of the CAT12 pipeline and used to statistically account for differences in head size.

© 2021 Moscoso A et al. *JAMA Neurology.*

eAppendix. Baseline Plasma P-Tau181 Associates With Baseline Neurodegeneration and Cognition

We investigated the associations between baseline plasma p-tau181 levels and baseline imaging markers of neurodegeneration. Analyses of FDG-PET and structural MRI data showed that, among CI participants, higher plasma p-tau181 levels were associated with both glucose hypometabolism and atrophy in highly AD-specific temporo-parietal spatial patterns (eFigure 2A and 2C). Correlations were stronger and spatially more extensive for hypometabolism compared to atrophy. No statistically significant associations were found among CU individuals. Compared to plasma p-tau181, plasma NfL displayed similar spatial associations with neurodegeneration in CI individuals (eFigure 2A and 2B) that, however, showed weaker correlations for FDG-PET (eFigure 2A). Furthermore, plasma NfL levels correlated with hypometabolism, but not with atrophy, also among CU individuals in widespread brain regions that involved frontal and temporal areas and were less reminiscent of an AD-typical neurodegeneration pattern. Multivariable analyses demonstrated that, in the CI group, both plasma biomarkers were independently associated with neurodegeneration in AD-vulnerable ROIs, although associations with hypometabolism were slightly stronger for plasma p-tau181 (eFigure 2C and 2D).

Regarding associations with baseline cognitive measures, baseline plasma p-tau181 levels were associated with poorer cognitive performance only in the CI group (CU: r=-0.02, *p*=0.69; CI: r=0.28, *p*<0.001). Similar results were obtained for plasma NfL (CU: r=-0.03, *p*=0.63; CI: r=0.22, *p*<0.001). In multivariable models, both plasma biomarkers were independently associated with cognition in the CI group, showing similar effect sizes (eFigure 3A). Approximately 50% of the strength of the association between plasma p-tau181 and cognition was mediated through neurodegeneration in AD-vulnerable areas (eFigure 3B), indicating links between plasma p-tau181 and cognition independent of imaging neurodegeneration markers.

The specificity of plasma p-tau181 for AD-related neurodegeneration suggested by the previous regional analyses was confirmed in analyses stratified by Aβ status: in the predefined Meta-ROIs, plasma p-tau181 was associated with hypometabolism and atrophy only in Aβ+ subjects (eFigure 4 and eFigure 5). Again, the voxel-wise patterns closely resembled AD-typical neurodegeneration patterns (eFigure 4). Similar results were found for associations with cognition: plasma p-tau181 correlated with poorer cognitive performance in CI Aβ+ subjects (Aβ+ CU: r=-0.14, *p*=0.13; Aβ+CI: r=0.23, *p*<0.001) but not in Aβ- (p>0.70). In contrast, plasma NfL showed significant associations with regional neurodegeneration markers across Aβ+ and Aβ- groups in both voxel-wise analyses and in Meta-ROIs of hypometabolism and atrophy (eFigure 6 and eFigure 7). Similarly, plasma NfL was associated with poorer cognition in CI Aβ+ (Aβ+ CU: r=-0.06, p=0.52; Aβ+CI: r=0.20, *p*<0.001) and CI Aβ- subjects (Aβ- CU: r=-0.02, *p*=0.81; Aβ- CI: r=0.16, *p*=0.02).

© 2021 Moscoso A et al. *JAMA Neurology.*

eFigure 1. Plasma P-Tau181 and Plasma NfL Distributions

Legend: Outliers were identified as those data points lying beyond 12 median absolute deviations (MAD) above the median. We used a more conservative criterium (4 times more stringent than the commonly used approach of 3 MADs above median) to minimally influence the dataset with outlier removal and exclude only the most extreme cases. We identified 4 outliers in the distribution of plasma p-tau181 and one in the distribution of plasma NfL (0.4% of the total sample). These outlier cases were highlighted with red circles. Maximum values of the outlier-removed distribution were also highlighted with red arrows.

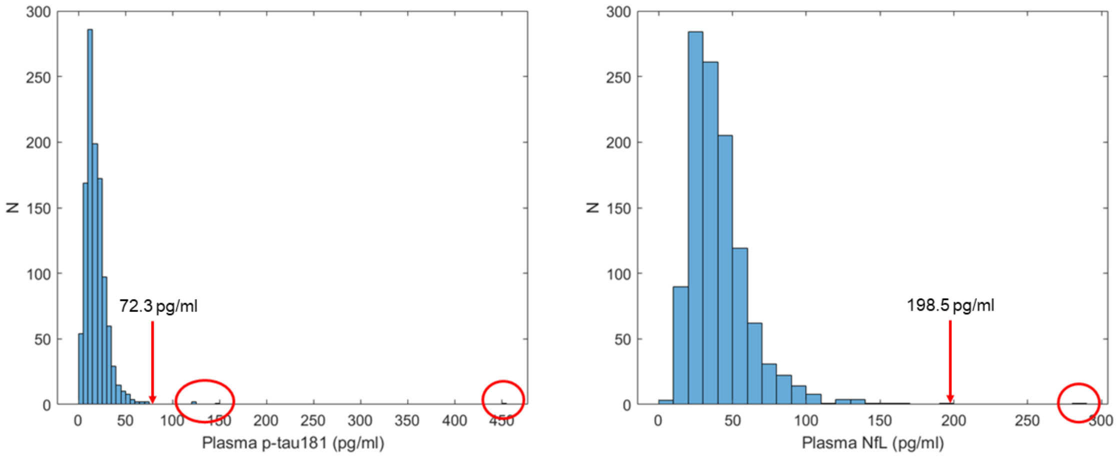

© 2021 Moscoso A et al. *JAMA Neurology.*

eFigure 2. Associations of Baseline Plasma P-Tau181 and NfL With Baseline Hypometabolism and Atrophy

Legend: Regression lines in all graphs were computed by setting covariates in the linear model to average group levels (CU or CI) and categorical variables to the reference (female sex and, for atrophy measures, 3T field strength). A-B) Age- and sex-adjusted associations of baseline plasma p-tau181 and NfL with baseline glucose hypometabolism (A) and gray matter atrophy (B) at the voxel- (upper row) and AD Meta-ROI-level (bottom row) in cognitively unimpaired and impaired individuals. In order to account for the difference in sample sizes, results of voxel-wise analyses were thresholded on the voxel-level at $p<0.01$ (uncorrected) for the CU group and $p<0.001$ (uncorrected) for CI. All maps were further thresholded using a family-wise error-corrected $p<0.05$ at the cluster level. C-D) Independents effects of baseline plasma p-tau181 and NfL on baseline glucose hypometabolism (C) and gray matter atrophy (D) in the AD Meta-ROI, estimated by simultaneously inputting them as independent variables in an age-, and sex- (and TIV and field strength for atrophy) adjusted linear regression. z-scores were computed using the respective group biomarker levels (CU or CI) as the reference. Reported standardized βs were computed using the covariate-adjusted linear models. CI above figure panels stands for cognitively impaired subjects.

© 2021 Moscoso A et al. *JAMA Neurology.*

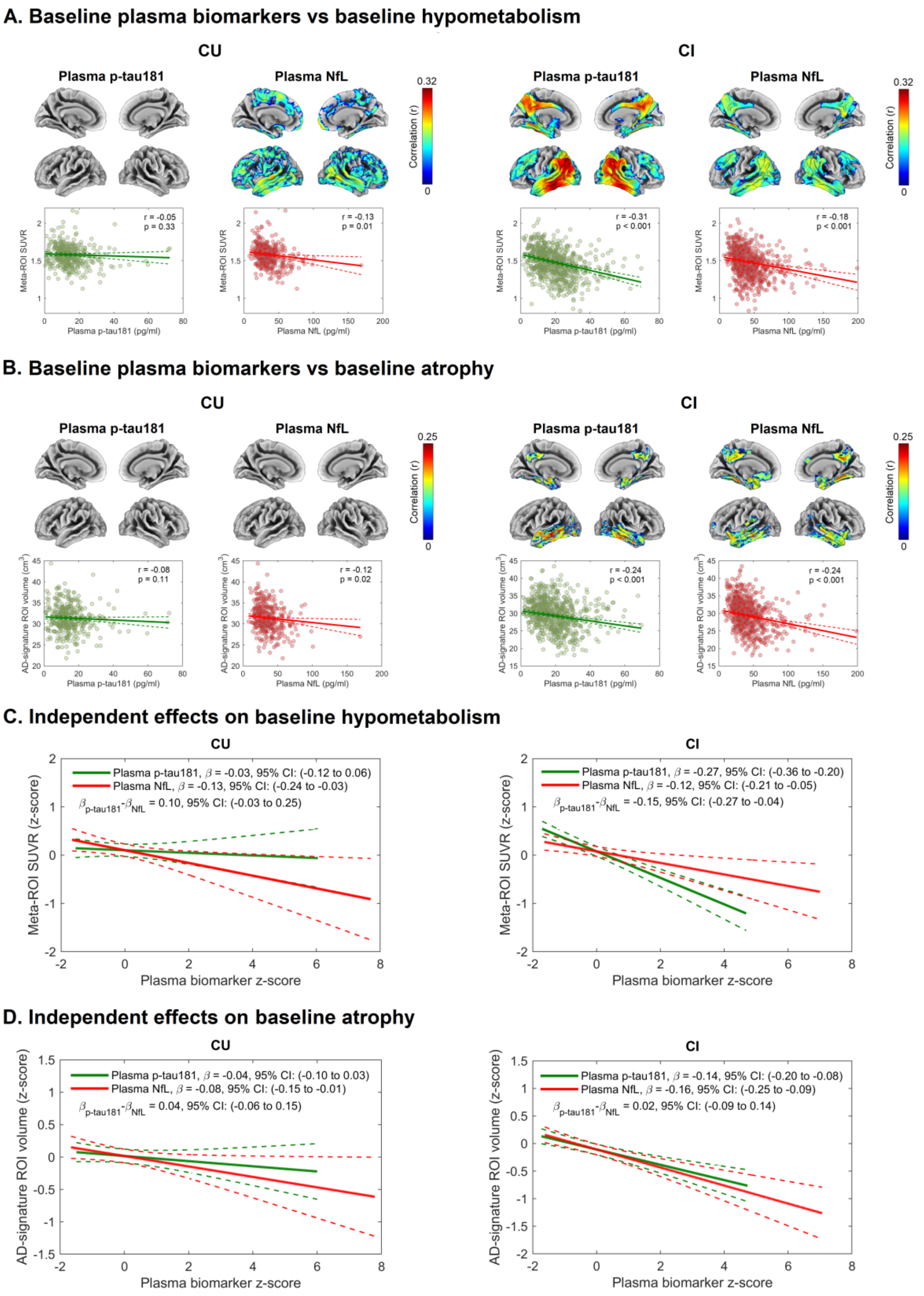

© 2021 Moscoso A et al. *JAMA Neurology.*

eFigure 3. Associations of Baseline Plasma Biomarker Levels With Baseline Cognition

Legend: A) Independents effects of baseline plasma p-tau181 and NfL on baseline PACC and ADAS-Cog 13, estimated by simultaneously inputting them as independent variables in an age-, sex-, and education-adjusted linear regressions. Reported partial correlation coefficients were adjusted for the same covariates. Regression lines were computed by setting covariates in the linear model to average group levels (CU or CI) and categorical variables to the reference (female sex). B) Mediation analyses testing whether imaging neurodegeneration markers (measured in AD-typical ROIs) mediated the associations between plasma p-tau181 and cognition. The mediated effect is reported when there is a statistically significant mediation, as it is designated as c-c′. The remaining effect of plasma p-tau181 on cognition after adjusting for the mediator is c′. The direct effect of plasma p-tau181 on the neurodegeneration markers is designated a, while the direct effect of these markers on cognition is b. CI above figure panels stands for cognitively impaired subjects.

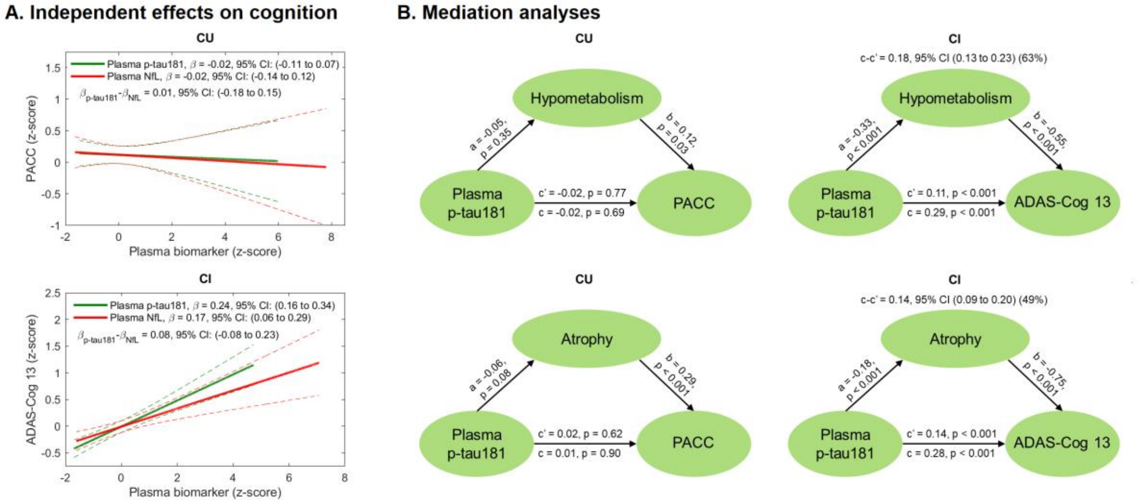

© 2021 Moscoso A et al. *JAMA Neurology.*

eFigure 4. Associations of Baseline Plasma P-Tau181 With Baseline Neurodegeneration Markers in Aβ+ Cognitively Unimpaired and Impaired Subjects

Legend: Associations of baseline plasma p-tau181 with baseline glucose hypometabolism (A) and gray matter atrophy (B) at the voxel and AD-ROI level. Statistical maps were thresholded using a lenient threshold ($p<0.05$ (uncorrected) at the voxel level and further thresholded at the cluster level by restricting results to clusters with a number of voxels higher than the expected number of voxels as predicted using random field theory) to maximize detection power in the Aβ- group while keeping identical thresholds for the Aβ+ group. Reported partial correlation coefficients were adjusted for the same covariates. Regression lines were computed by setting covariates in the linear model to average group levels (CU or CI) and categorical variables to the reference (female sex and, for atrophy measures, 3T field strength). CI stands for cognitively impaired subjects.

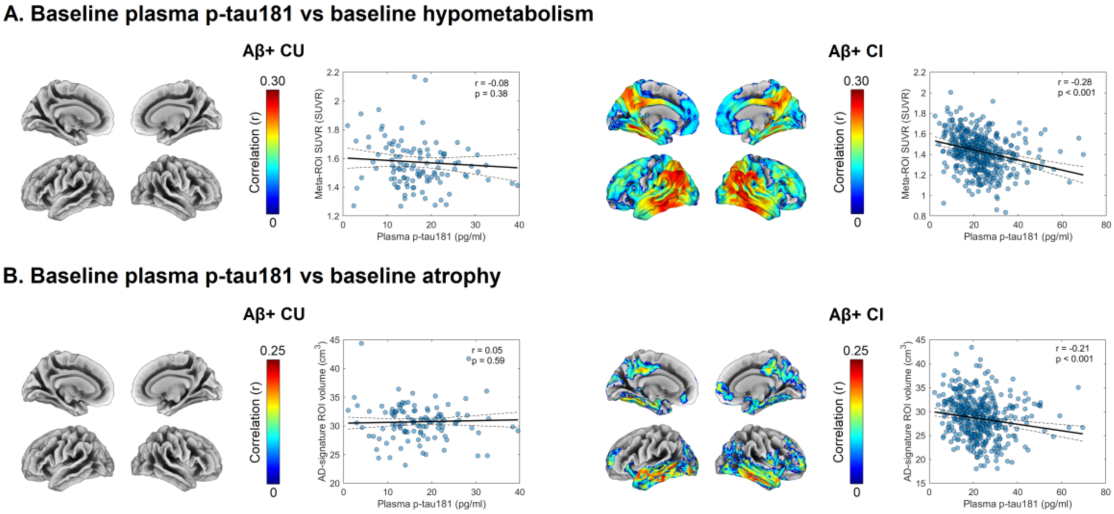

© 2021 Moscoso A et al. *JAMA Neurology.*

eFigure 5. Associations of Baseline Plasma P-Tau181 With Baseline Neurodegeneration Markers in Aβ- Cognitively Unimpaired and Impaired Subjects

Legend: Reported partial correlation coefficients were adjusted for age, sex, and, for atrophy measures, TIV and field strength. Regression lines were computed by setting covariates in the linear model to average group levels (CU or CI) and categorical variables to the reference (female sex and, for atrophy measures, 3T field strength). No statistically significant associations were observed at the voxel level (uncorrected *p*<0.05 at the voxel level, number of voxels per cluster > expected number of voxels per cluster according to random field theory). CI stands for cognitively impaired subjects.

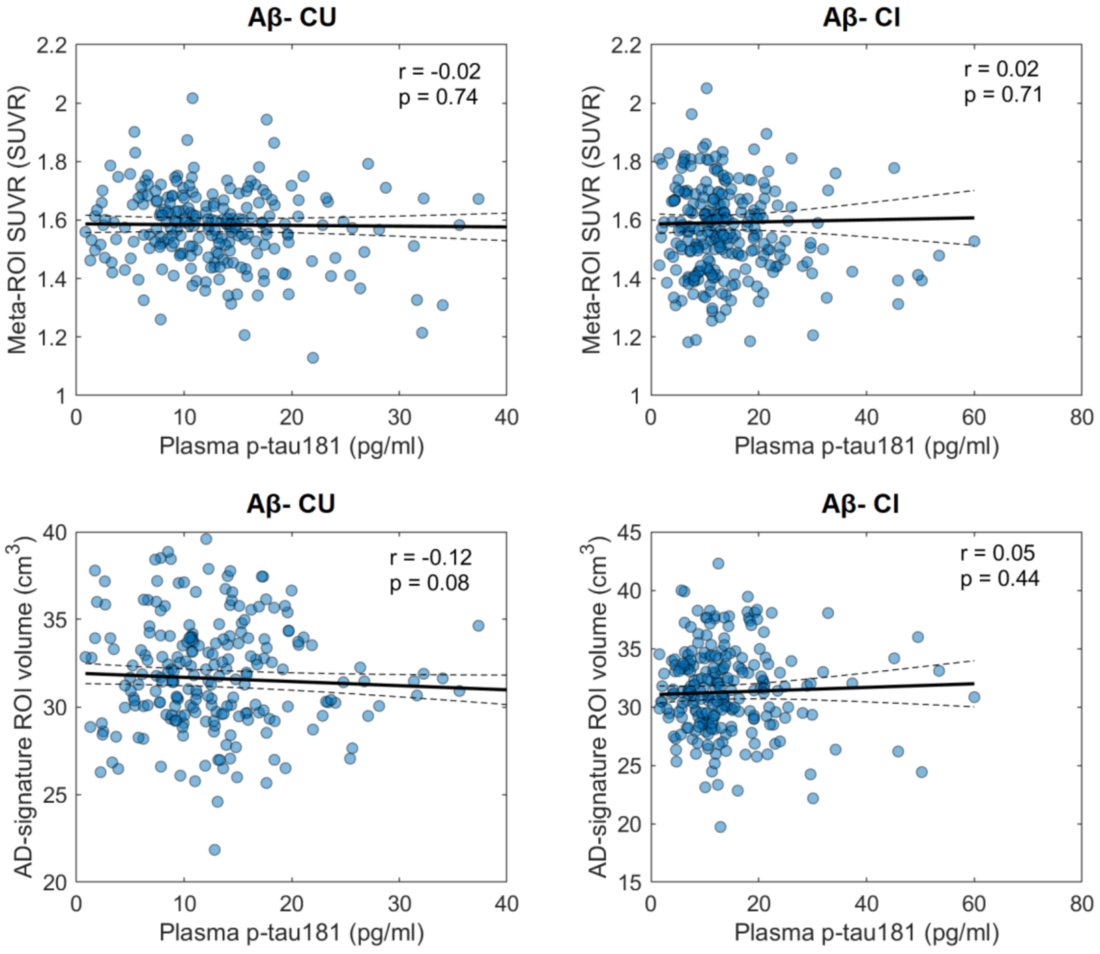

© 2021 Moscoso A et al. *JAMA Neurology.*

eFigure 6. Associations of Baseline Plasma NfL With Baseline Neurodegeneration Markers in Aβ- Cognitively Unimpaired and Impaired Subjects

Legend: Associations of baseline plasma NfL with baseline glucose hypometabolism (A) and gray matter atrophy (B) at the voxel and AD-ROI level. Models were adjusted for age, sex, and, for atrophy measures, TIV and field strength. Statistical maps were thresholded using a lenient threshold (*p*<0.05 (uncorrected) at the voxel level and further thresholded at the cluster level by restricting results to clusters with a number of voxels higher than the expected number of voxels as predicted using random field theory) to maximize detection power in the Aβ- group. Reported partial correlation coefficients were adjusted for the same covariates. Regression lines were computed by setting covariates in the linear model to average group levels (CU or CI) and categorical variables to the reference (female sex and, for atrophy measures, 3T field strength). CI stands for cognitively impaired subjects.

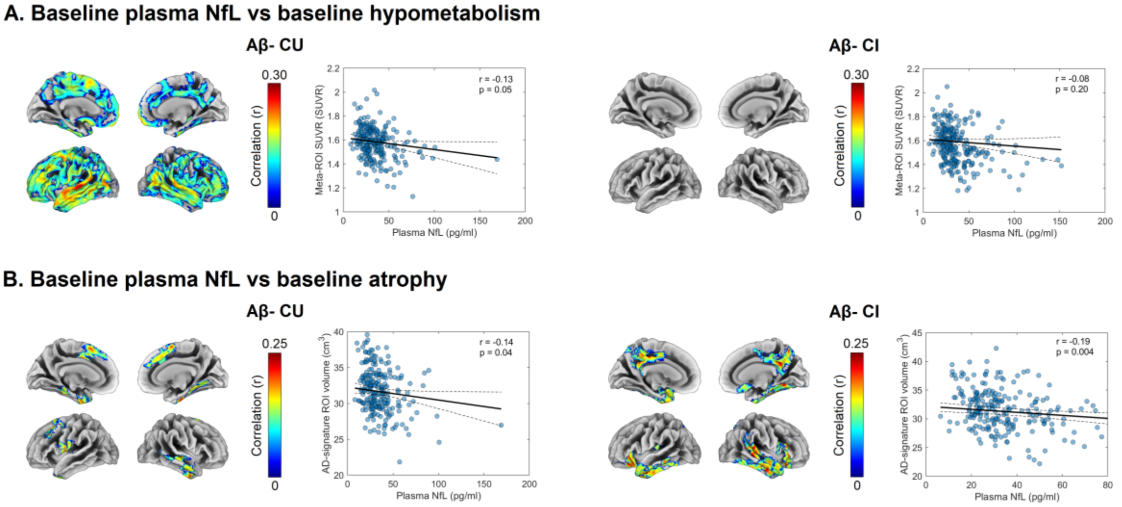

© 2021 Moscoso A et al. *JAMA Neurology.*

eFigure 7. Associations of Baseline Plasma NfL With Baseline Neurodegeneration Markers in Aβ+ Cognitively Unimpaired and Impaired Subjects

Legend: Associations of baseline plasma NfL with baseline glucose hypometabolism (A) and gray matter atrophy (B) at the voxel and AD-ROI level. Models were adjusted for age, sex, and, for atrophy measures, TIV and field strength. Statistical maps were thresholded using a lenient threshold (p<0.05 (uncorrected) at the voxel level and further thresholded at the cluster level by restricting results to clusters with a number of voxels higher than the expected number of voxels as predicted using random field theory) to maximize detection power in the Aβ- group while keeping identical thresholds for the Aβ+ group. Reported partial correlation coefficients were adjusted for the same covariates. Regression lines were computed by setting covariates in the linear model to average group levels (CU or CI) and categorical variables to the reference (female sex and, for atrophy measures, 3T field strength). CI stands for cognitively impaired subjects.

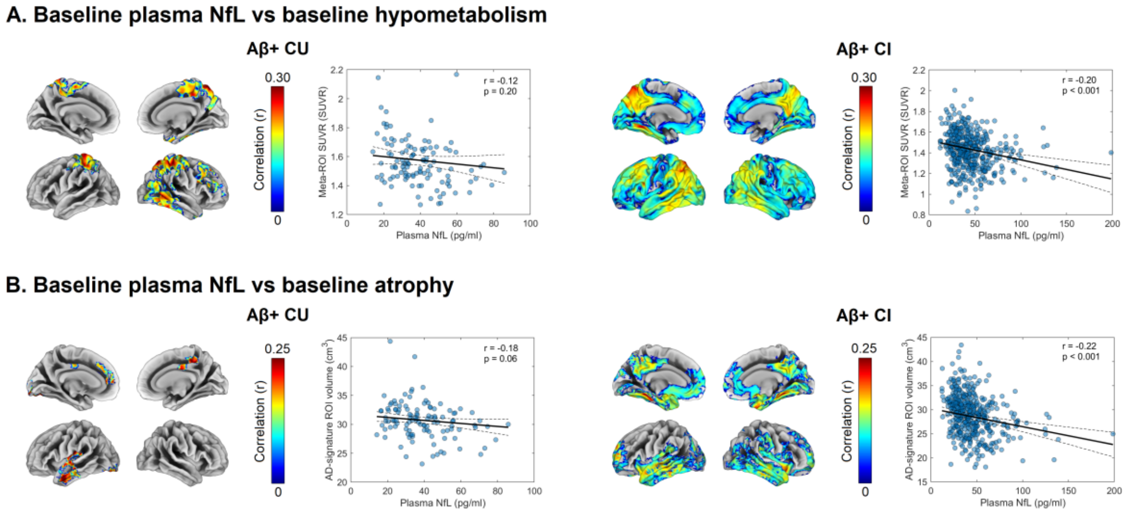

© 2021 Moscoso A et al. *JAMA Neurology.*

eFigure 8. Typical Spatial Patterns of Hypometabolism and Atrophy in AD

Legend: Average z-score maps (using the Aβ- CN sample as the reference) of brain metabolism and regional volume in Aβ+ AD dementia patients from our study cohort. Lower z-scores represent lower glucose metabolism and greater atrophy.

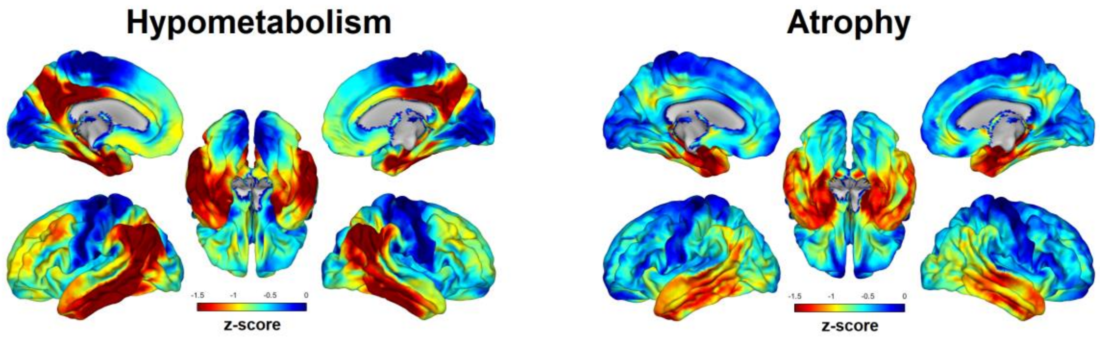

© 2021 Moscoso A et al. *JAMA Neurology.*

eFigure 9. Brain Regions in Which Baseline Plasma P-Tau181 and Plasma NfL Correlate With Longitudinal Decrease in Glucose Metabolism and Greater Gray Matter Atrophy

Legend: Spatial overlap (depicted in green) of the association patterns of baseline plasma p-tau181 (blue) and plasma NfL (orange) with longitudinally A) decreasing glucose metabolism and B) increasing GM atrophy. CI stands for cognitively impaired subjects.

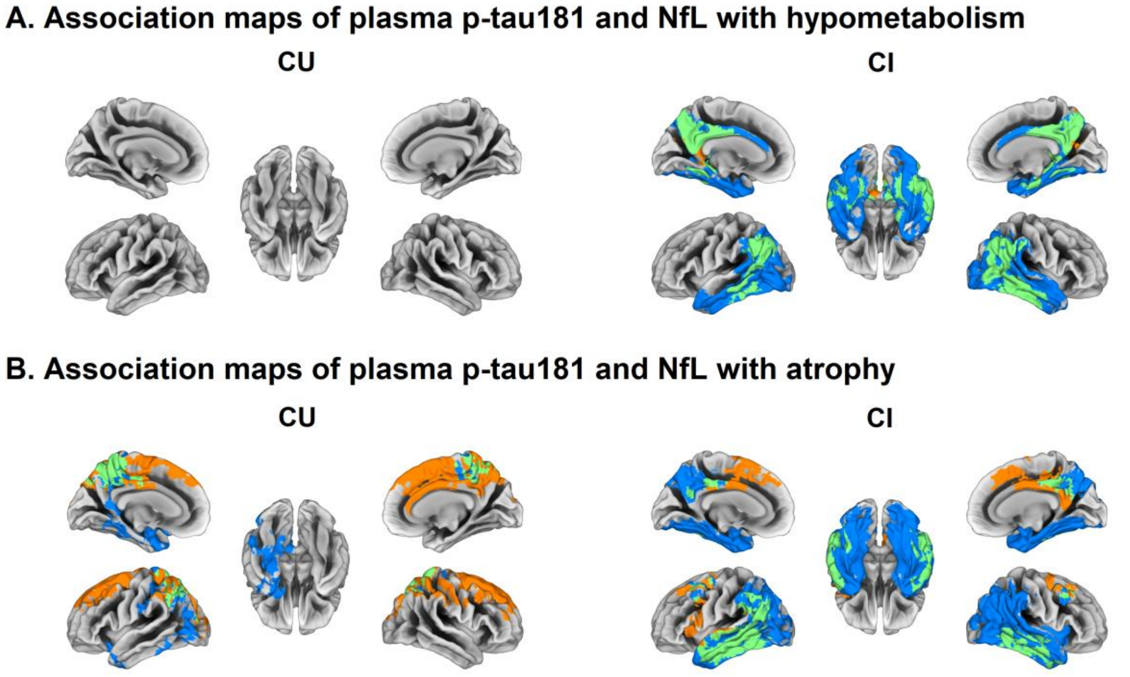

© 2021 Moscoso A et al. *JAMA Neurology.*

eFigure 10. Associations Between Plasma P-Tau181 and Plasma NfL

Legend: Reported partial correlation coefficients were adjusted for age and sex. Regression lines were computed by setting covariates in the linear model to average group levels (CU or CI) and categorical variables to the reference (female sex). CI stands for cognitively impaired subjects.

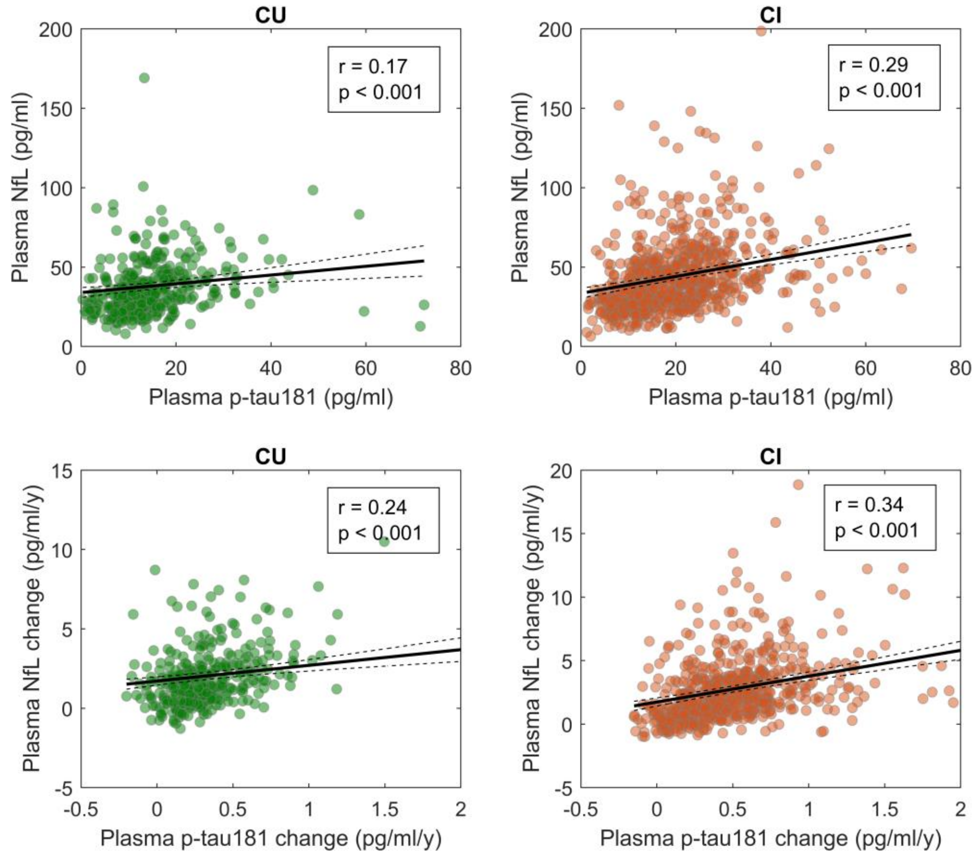

© 2021 Moscoso A et al. *JAMA Neurology.*

eFigure 11. Independent Effects of Baseline Plasma Biomarkers on Longitudinal Neurodegeneration

Legend: Independents effects of baseline plasma p-tau181 and NfL on hypometabolism (A) and (B) atrophy progression in AD-typical ROIs, estimated by using both plasma biomarkers as independent variables in a combined linear regression model adjusted for age, and sex (as well as TIV and field strength for atrophy). z-scores were computed using the respective group (CU or CI) biomarker (or biomarker change) levels as the reference. Reported standardized β coefficients were computed using the covariate-adjusted linear models. Regression lines were computed by setting covariates in the linear model to average group levels (CU or CI) and categorical variables to the reference (female sex and, for atrophy measures, 3T field strength). CI above figure panels stands for cognitively impaired subjects.

A. Independent effects on hypometabolism progression

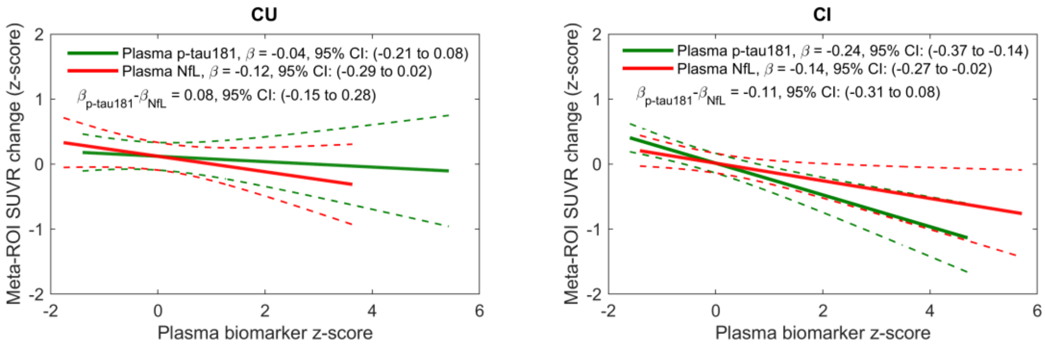

B. Independent effects on atrophy progression

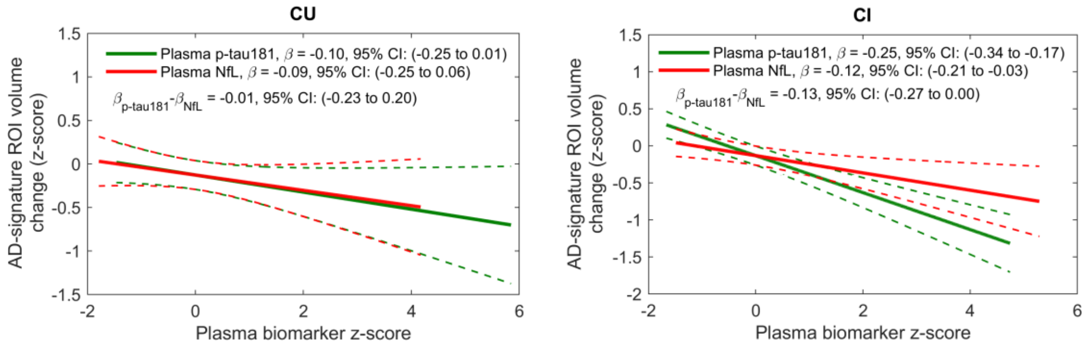

© 2021 Moscoso A et al. *JAMA Neurology.*

eFigure 12. Associations of Baseline Plasma Biomarker Levels With Cognitive Decline

Legend: A) Independents effects of baseline plasma p-tau181 and NfL on PACC change (for CU participants) and ADAS-Cog 13 change (for CI participants), estimated by using both plasma biomarkers as independent variables in a combined linear regression model adjusted for age, sex, and education. z-scores were computed using the respective group (CU or CI) biomarker (or biomarker change) levels as the reference. Reported standardized β coefficients were computed using the covariate-adjusted linear models. Regression lines were computed by setting covariates in the linear model to average group levels (CU or CI) and categorical variables to the reference (female sex). B) Mediation analyses testing whether imaging neurodegeneration markers (measured in AD-typical ROIs) mediated the associations between plasma p-tau181 and cognition. The mediated effect is reported when there is a statistically significant mediation, as estimated by the difference between the total (c) and direct (c’) effect of plasma p-tau181 on cognitive change (c-c′). The direct effect of plasma p-tau181 on cognition (c’) is estimated by adjusting the association between plasma p-tau181 and cognitive change (total effect, c) for the mediator. The direct effect of plasma p-tau181 on the neurodegeneration markers is designated a, while the direct effect of these markers on cognition is b. CI above figure panels stands for cognitively impaired subjects.

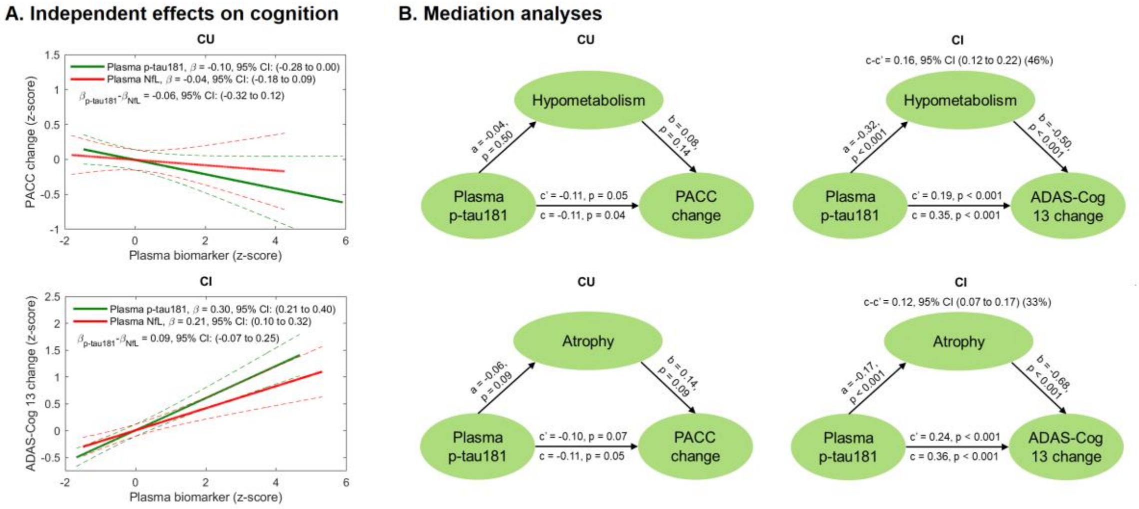

© 2021 Moscoso A et al. *JAMA Neurology.*

eFigure 13. Associations of Baseline Plasma P-Tau181 With Longitudinal Neurodegeneration Markers in Aβ- Cognitively Unimpaired and Impaired Subjects

Legend: Reported partial correlation coefficients were adjusted for age, sex, and, for atrophy measures, TIV and field strength. Regression lines were computed by setting covariates in the linear model to average group levels (CU or CI) and categorical variables to the reference (female sex and, for atrophy measures, 3T field strength). No statistically significant associations were observed at the voxel level (uncorrected $p<0.05$ at the voxel level, number of voxels per cluster > expected number of voxels per cluster according to random field theory). CI stands for cognitively impaired subjects.

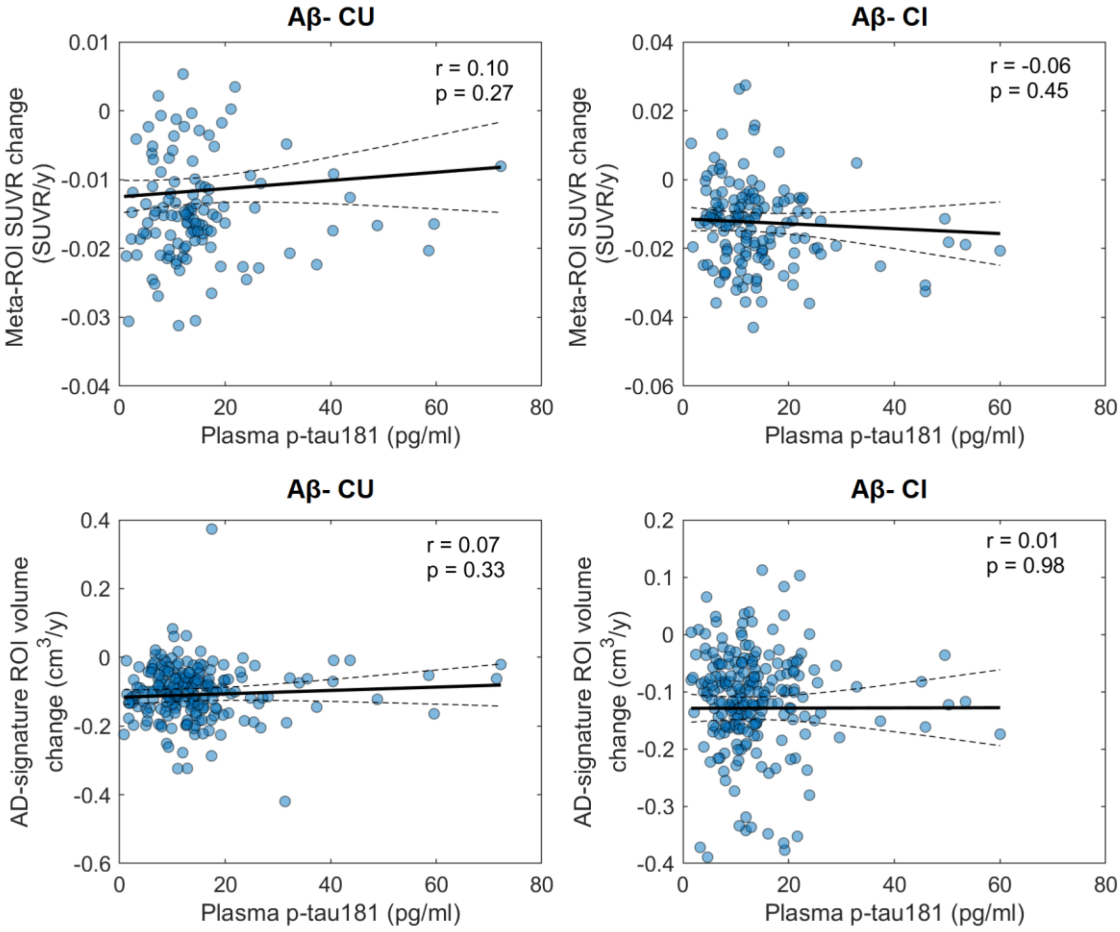

© 2021 Moscoso A et al. *JAMA Neurology.*

eFigure 14. Associations of Baseline Plasma NfL With Longitudinal Neurodegeneration Markers in Aβ- Cognitively Unimpaired and Impaired Subjects

Legend: Associations of baseline plasma NfL with longitudinal glucose hypometabolism (A) and gray matter atrophy (B) at the voxel and ROI level. Models were adjusted for age, sex, and, for atrophy measures, TIV and field strength. Reported partial correlation coefficients were adjusted for the same covariates. Statistical maps were thresholded using a lenient threshold (p<0.05 (uncorrected) at the voxel level and further thresholded at the cluster level by restricting results to clusters with a number of voxels higher than the expected number of voxels as predicted using random field theory) to maximize detection power in the Aβ- group. Regression lines for ROI analyses were computed by setting covariates in the linear model to average group levels (CU or CI) and categorical variables to the reference (female sex and, for atrophy measures, 3T field strength). CI stands for cognitively impaired subjects.

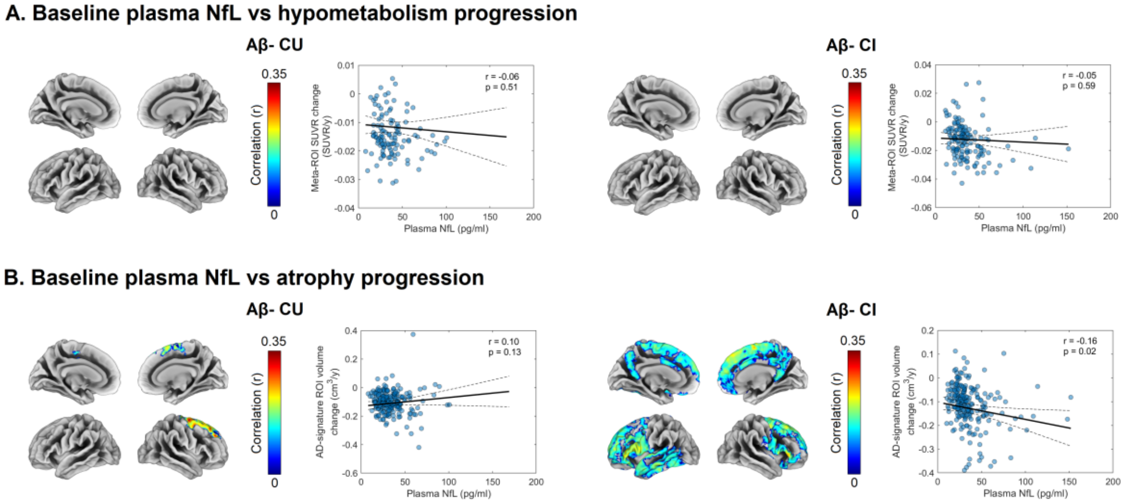

© 2021 Moscoso A et al. *JAMA Neurology.*

eFigure 15. Associations of Baseline Plasma NfL With Longitudinal Neurodegeneration Markers in Aβ+ Cognitively Unimpaired and Impaired Subjects

Legend: Associations of baseline plasma NfL with longitudinal glucose hypometabolism (A) and gray matter atrophy (B) at the voxel and ROI level. Models were adjusted for age, sex, and, for atrophy measures, TIV and field strength. Reported partial correlation coefficients were adjusted for the same covariates. Statistical maps were thresholded using a lenient threshold (uncorrected *p*<0.05 at the voxel level and further thresholded at the cluster level by restricting to clusters with a number of voxels higher than the expected number of voxels as predicted using random field theory) to maximize detection power in the Aβ- group while keeping identical thresholds for the Aβ+ group. Reported partial correlation coefficients were adjusted for the same covariates. Regression lines were computed by setting covariates in the linear model to average group levels (CU or CI) and categorical variables to the reference (female sex and, for atrophy measures, 3T field strength). C) and D): Spatial overlap (depicted in green) of the association patterns of baseline plasma p-tau181 (blue) and plasma NfL (orange) with longitudinally A) decreasing glucose metabolism and B) increasing GM atrophy. CI stands for cognitively impaired subjects.

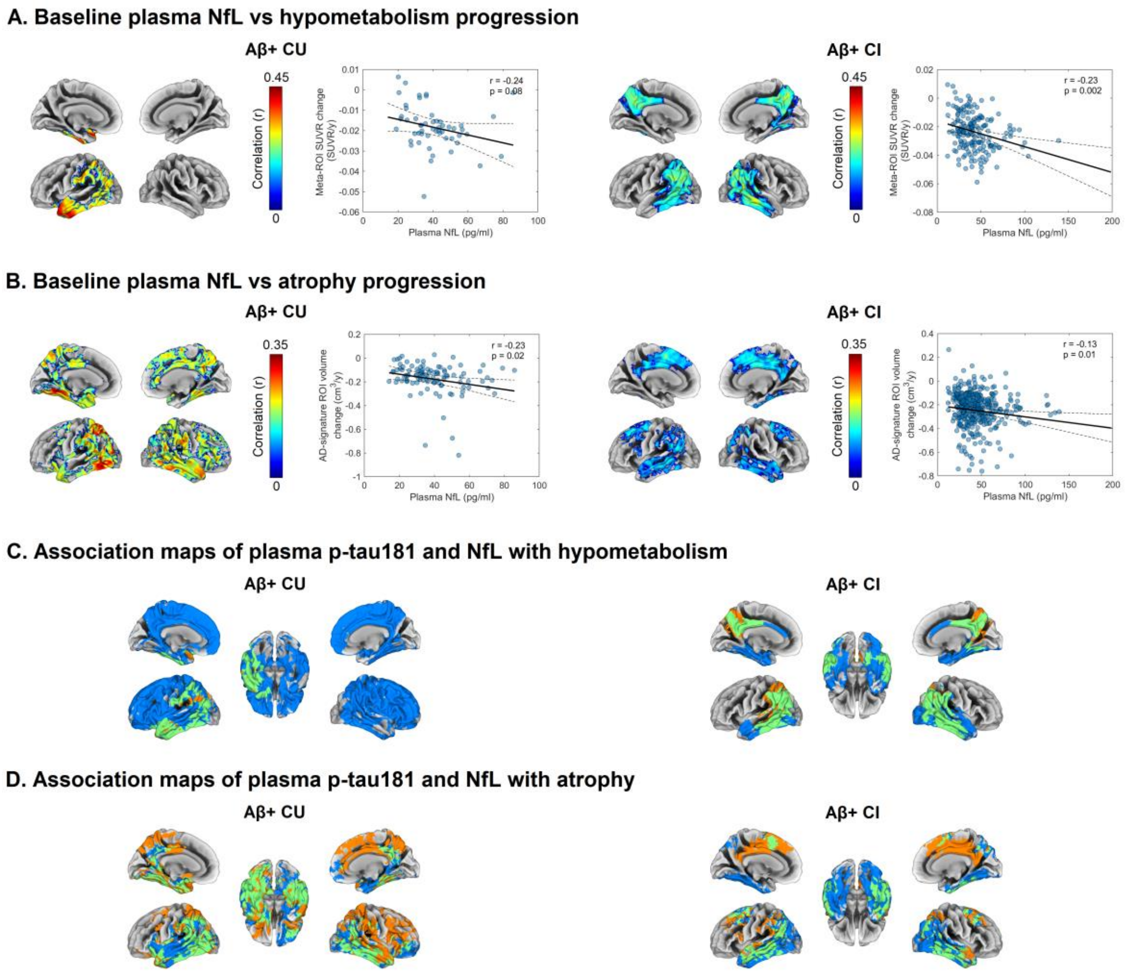

© 2021 Moscoso A et al. *JAMA Neurology.*

eFigure 16. Brain Regions in Which Longitudinal Plasma P-Tau181 and Plasma NfL Correlate With Longitudinal Decrease in Glucose Metabolism and Greater Gray Matter Atrophy

Legend: Spatial overlap (depicted in green) of the association patterns of longitudinal plasma p-tau181 (blue) and plasma NfL (orange) with longitudinally A) decreasing glucose metabolism and B) increasing GM atrophy. CI stands for cognitively impaired subjects.

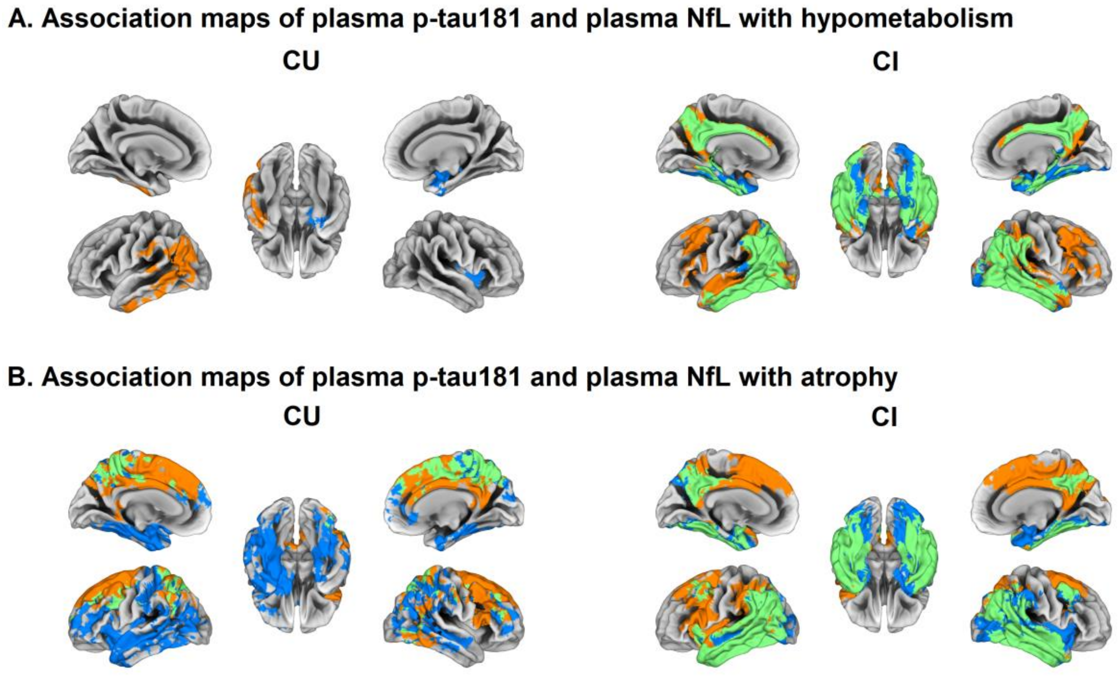

© 2021 Moscoso A et al. *JAMA Neurology.*

eFigure 17. Independent Effects of Plasma Biomarker Change on Longitudinal Neurodegeneration

Legend: Independents effects of plasma p-tau181 and NfL longitudinal changes on hypometabolism (A) and atrophy progression (B) in AD-typical ROIs, estimated by using both plasma biomarkers as independent variables in a combined linear regression model adjusted for age, sex, and, for atrophy measures, TIV and field strength. z-scores were computed using the respective group (CU or CI) biomarker (or biomarker change) levels as the reference. Reported standardized β coefficients were computed using the covariate-adjusted linear models. Regression lines were computed by setting covariate values in the linear model to average group levels (CU or CI) and categorical variables to the reference (female sex and, for atrophy measures, 3T field strength). CI above figure panels stands for cognitively impaired subjects.

A. Independent effects on hypometabolism progression

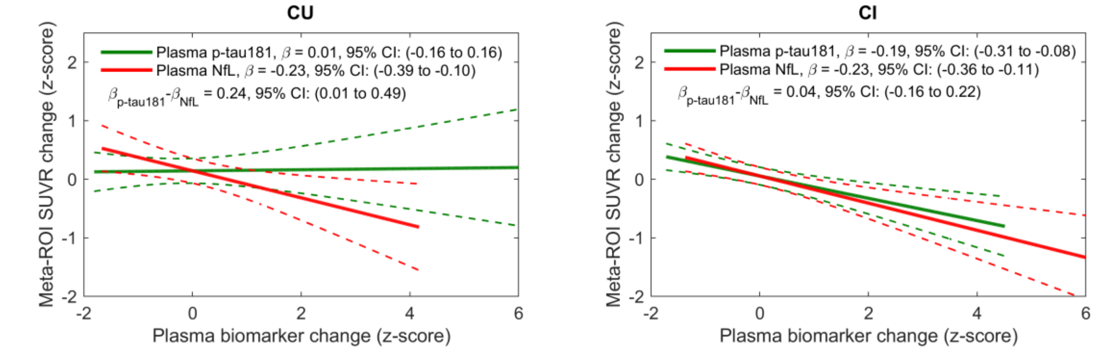

B. Independent effects on atrophy progression

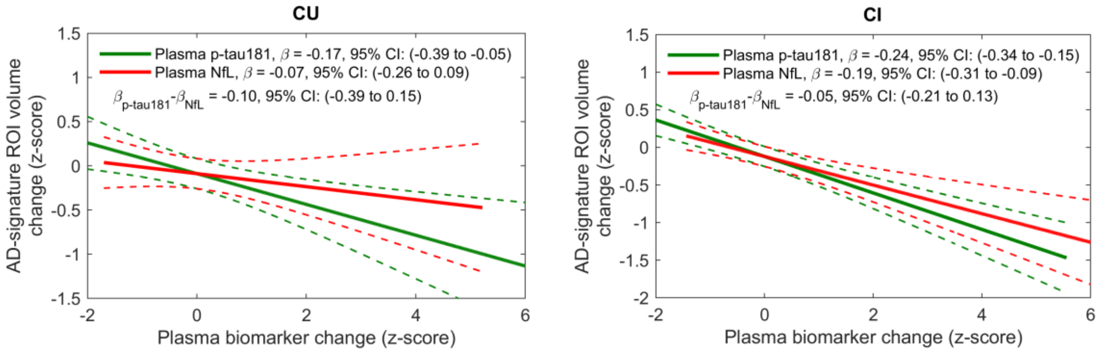

© 2021 Moscoso A et al. *JAMA Neurology.*

eFigure 18. Associations of Plasma Biomarker Longitudinal Change With Cognitive Decline

Legend: A) Independents effects of plasma p-tau181 and NfL longitudinal change on PACC change (for CU participants) and ADAS-Cog 13 change (for CI participants), estimated by using both plasma biomarkers as independent variables in a combined linear regression model adjusted for age, sex, and education. z-scores were computed using the respective group (CU or CI) biomarker (or biomarker change) levels as the reference. Reported standardized β coefficients were computed using the covariate-adjusted linear models. Regression lines were computed by setting covariates in the linear model to average group levels (CU or CI) and categorical variables to the reference (female sex). B) Mediation analyses testing whether imaging neurodegeneration markers (measured in AD-typical ROIs) mediated the associations between plasma p-tau181 and cognition. The mediated effect is reported when there is a statistically significant mediation, as estimated by the difference between the total (c) and direct (c') effect of plasma p-tau181 on cognitive change (c-c′). The direct effect of plasma p-tau181 on cognition (c') is estimated by adjusting the association between plasma p-tau181 and cognitive change (total effect, c) for the mediator. The direct effect of plasma p-tau181 on the neurodegeneration markers is designated a, while the direct effect of these markers on cognition is b. CI above figure panels stands for cognitively impaired subjects.

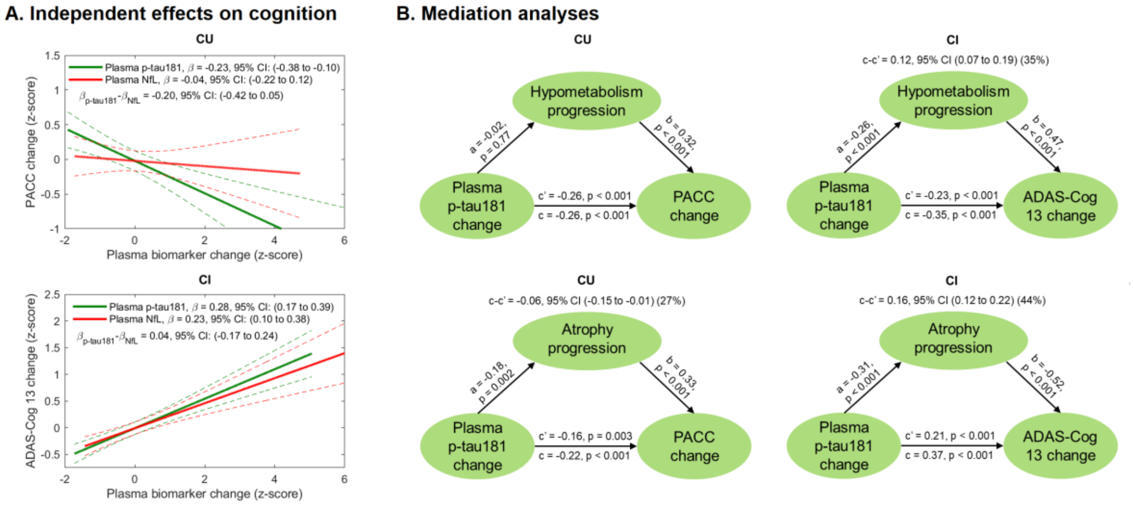

© 2021 Moscoso A et al. *JAMA Neurology.*

eFigure 19. Associations of Plasma P-Tau181 Longitudinal Change With Longitudinal Neurodegeneration Markers in Aβ- Cognitively Unimpaired and Impaired Subjects

Legend: Reported partial correlation coefficients were adjusted for age, sex, and, for atrophy measures, for TIV and field strength. Regression lines were computed by setting covariates in the linear model to average group levels (CU or CI) and categorical variables to the reference (female sex and, for atrophy measures, 3T field strength). No statistically significant associations were observed at the voxel level (uncorrected *p*<0.05 at the voxel level, number of voxels per cluster > expected number of voxels per cluster according to random field theory). CI stands for cognitively impaired subjects.

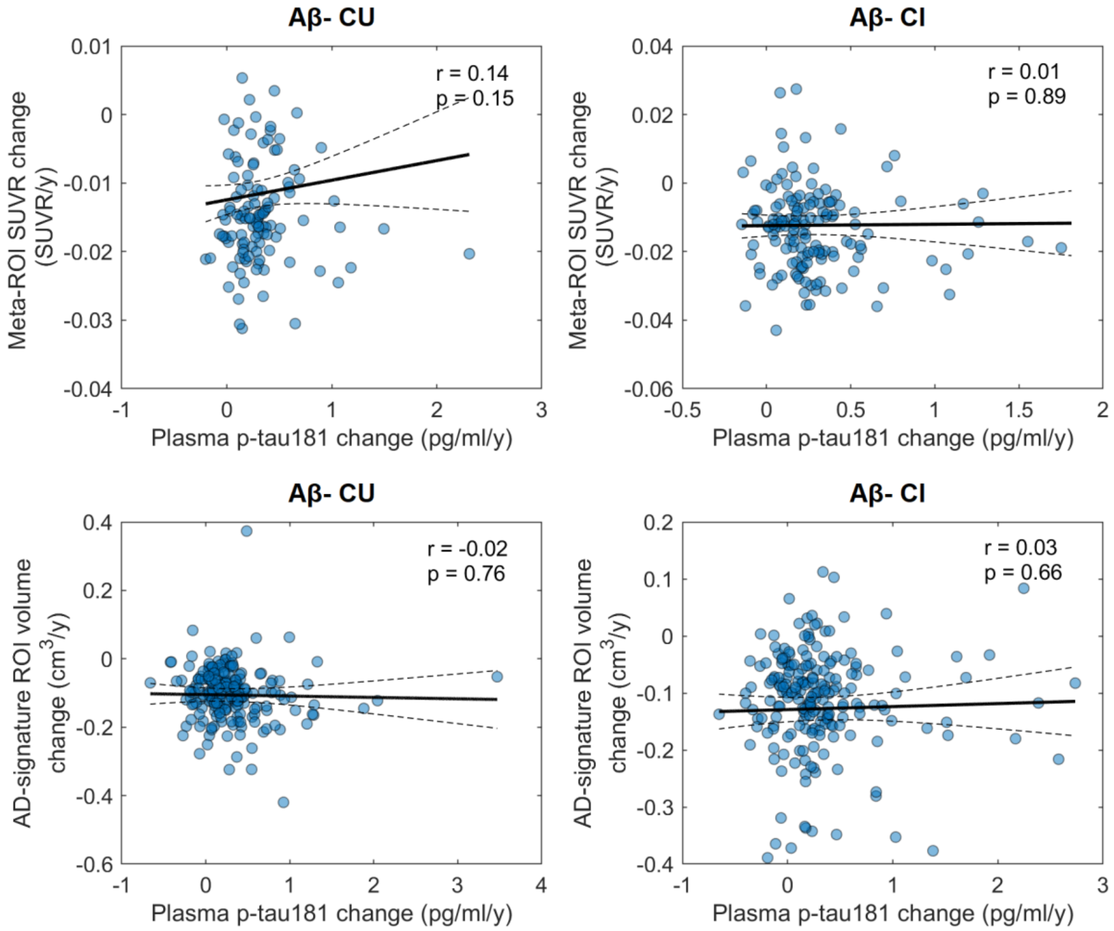

© 2021 Moscoso A et al. *JAMA Neurology.*

eFigure 20. Associations of Plasma NfL Longitudinal Change With Longitudinal Neurodegeneration Markers in Aβ- Cognitively Unimpaired and Impaired Subjects

Legend: Associations of plasma NfL longitudinal change with longitudinal glucose hypometabolism (A) and gray matter atrophy (B) at the voxel and ROI level. Models were adjusted for age, sex, and, for atrophy measures, TIV and field strength. Statistical maps were thresholded using a lenient threshold (uncorrected p<0.05 at the voxel level and further thresholded at the cluster level by restricting to clusters with a number of voxels higher than the expected number of voxels as predicted using random field theory) to maximize detection power in the Aβ- group. Reported partial correlation coefficients were adjusted for the same covariates. Regression lines were computed by setting covariates in the linear model to average group levels (CU or CI) and categorical variables to the reference (female sex and, for atrophy measures, 3T field strength). CI stands for cognitively impaired subjects.

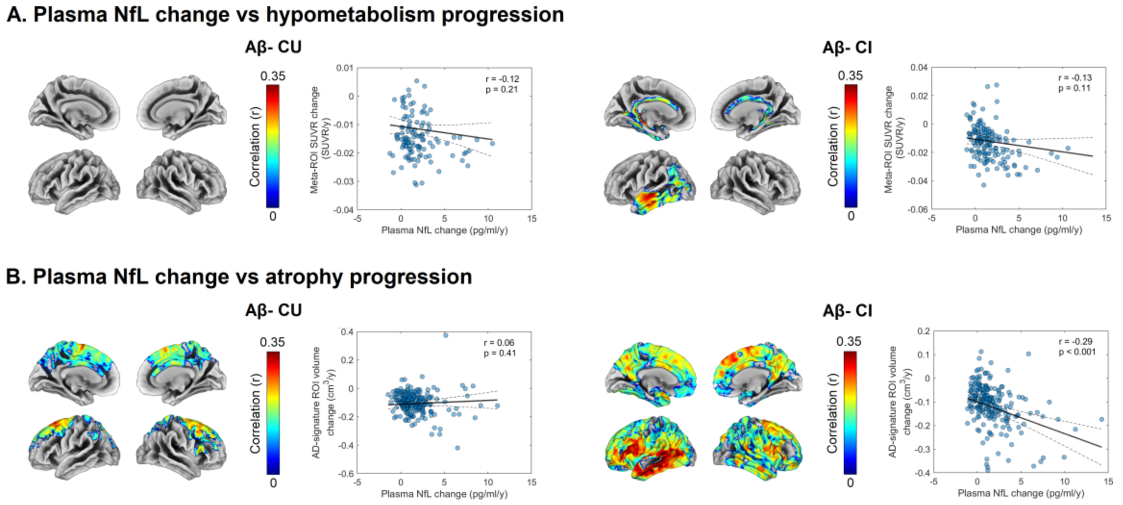

© 2021 Moscoso A et al. *JAMA Neurology.*

eFigure 21. Associations of Plasma NfL Longitudinal Change With Longitudinal Neurodegeneration Markers and Spatial Overlap With Plasma P-Tau181 in Aβ+ Cognitively Unimpaired and Impaired Subjects

Legend: Associations of plasma NfL longitudinal change with longitudinal glucose hypometabolism (A) and gray matter atrophy (B) at the voxel and ROI level. Models were adjusted for age, sex, and, for atrophy measures, TIV and field strength. Statistical maps were thresholded using a lenient threshold (uncorrected *p*<0.05 at the voxel level and further thresholded at the cluster level by restricting to clusters with a number of voxels higher than the expected number of voxels as predicted using random field theory) to maximize detection power in the Aβ- group while keeping identical thresholds for the Aβ+ group. Reported partial correlation coefficients were adjusted for the same covariates. Regression lines were computed by setting covariates in the linear model to average group levels (CU or CI) and categorical variables to the reference (female sex and, for atrophy measures, 3T field strength). C) and D): Spatial overlap (depicted in green) of the association patterns of longitudinal plasma p-tau181 (blue) and plasma NfL (orange) with longitudinal C) hypometabolism and D) atrophy. CI stands for cognitively impaired subjects.

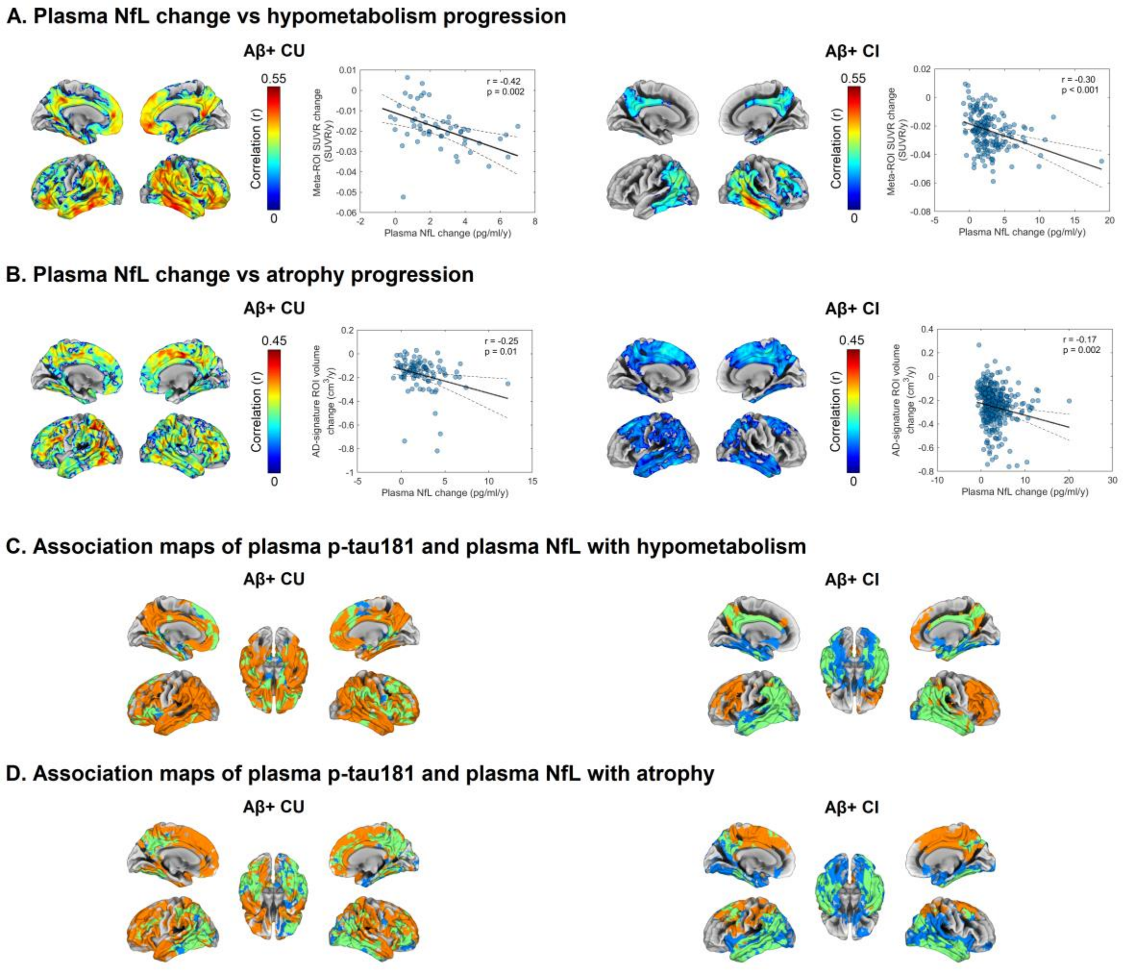

© 2021 Moscoso A et al. *JAMA Neurology.*

eTable. Associations of Plasma P-Tau181 and Plasma NfL With Hippocampus Volumes

Legend: We reported partial correlation coefficients (r) adjusted for age, sex, TIV, and field strength for 1) baseline plasma biomarkers vs hippocampus volume, 2) baseline plasma biomarkers vs longitudinal hippocampus volume change, and 3) plasma biomarkers change vs longitudinal hippocampus volume change. CI stands for cognitively impaired subjects.

	**CU**	**CI**	**Aβ- CU**	**Aβ+ CU**	**Aβ- CI**	**Aβ+ CI**
**Analysis**						
Bl p-tau181 vs Bl Hippo	-0.01	-0.21***	0.01	-0.08	-0.04	-0.18***
Bl NfL vs Bl Hippo	-0.02	-0.18***	-0.02	-0.06	-0.14*	-0.16***
Bl p-tau181 vs Hippo change	-0.18**	-0.33***	-0.07	-0.35***	-0.06	-0.25***
Bl NfL vs Bl Hippo change	-0.17**	-0.24***	-0.06	-0.26**	-0.16*	-0.22***
p-tau181 change vs Hippo change	-0.18**	-0.32***	-0.18*	-0.24*	-0.09	-0.26***
NfL change vs Bl Hippo change	-0.20***	-0.33***	-0.08	-0.34***	-0.30***	-0.27***

**p*<0.05; ***p*<0.01; ****p*<0.001

© 2021 Moscoso A et al. *JAMA Neurology.*

eReferences

1. Lange C, Suppa P, Frings L, Brenner W, Spies L, Buchert R. Optimization of Statistical Single Subject Analysis of Brain FDG PET for the Prognosis of Mild Cognitive Impairment-to-Alzheimer's Disease Conversion. *J Alzheimers Dis.* 2016;49(4):945-959.
2. Ashburner J, Ridgway GR. Symmetric diffeomorphic modeling of longitudinal structural MRI. *Front Neurosci.* 2012;6:197.

© 2021 Moscoso A et al. *JAMA Neurology.*
