# Supplementary material for: Longitudinal Associations of Blood Phosphorylated Tau181 and Neurofilament Light Chain With Neurodegeneration in Alzheimer Disease
Source: JAMA Neurol. 2021 Jan 11;78(4):1–12. doi: 10.1001/jamaneurol.2020.4986 (PMC7802009; doi:10.1001/jamaneurol.2020.4986)
Supplement: Supplement 2. — Nonauthor Collaborators [file jamaneurol-e204986-s002.pdf]

Supplemental Online Content: Nonauthor Collaborators

*Indicates required information. Only first name, last name, and suffix will appear in PubMed.

Group Name:* Alzheimer's Disease Neuroimaging Initiative							
First Name, Middle Initial*	Last Name*	Suffix*	Academic Degrees	Institution	Location (city, state/province, country)	Role or Contribution, eg, chair, principal investigator	Subgroup, eg, Steering Committee
Michael W.	Weiner		MD				
Paul	Aisen		MD				
Ronald	Petersen		MD, PhD				
Clifford R.	Jack, Jr.		MD				
William	Jagust		MD				
John Q.	Trojanowki		MD, PhD				
Arthur W.	Toga		PhD				
Laurel	Beckett		PhD				
Robert C.	Green		MD, MPH				
Andrew J.	Saykin		PsyD				
John	Morris		MD				
Leslie M.	Shaw		PhD				
Zaven	Khachaturian		PhD				
Greg	Sorensen		MD				
Maria	Carrillo		PhD				
Lew	Kuller		MD				
Marc	Raichle		MD				
Steven	Paul		MD				
Peter	Davies		MD				
Howard	Fillit		MD				
Franz	Hefti		PhD				
David	Holtzman		MD				
M. Marcel	Mesulam		MD				
William	Potter		MD				
Peter	Snyder		PhD				
Veronika	Logovinsky		MD, PhD				
Tom	Montine		MD, PhD				
Gustavo	Jimenez		MBS				
Michael	Donohue		PhD				
Devon	Gessert		BS				

Supplemental Online Content: Nonauthor Collaborators

*Indicates required information. Only first name, last name, and suffix will appear in PubMed.

First Name, Middle Initial*	Last Name*	Suffix*	Academic Degrees	Institution	Location (city, state/province, country)	Role or Contribution, eg, chair, principal investigator	Subgroup, eg, Steering Committee
Kelly	Harless		BA				
Jennifer	Salazar		MBS				
Yuliana	Cabrera		BS				
Sarah	Walter		MSc				
Lindsey	Hergesheimer		BS				
Danielle	Harvey		PhD				
Matthew	Bernstein		PhD				
Nick	Fox		MD				
Paul	Thompson		PhD				
Norbert	Schuff		PhD				
Charles	DeCarli		MD				
Bret	Borowski		RT				
Jeff	Gunter		PhD				
Matt	Senjem		MS				
Prashanthi	Vemuri		PhD				
David	Jones		MD				
Kejal	Kantarci		MD				
Chad	Ward						
Robert A.	Koeppe		PhD				
Norm	Foster		MD				
Eric M.	Reiman,		MD				
Kewei	Chen		PhD				
Chet	Mathis		MD				
Susan	Landau		PhD				
John C.	Morris		MD				
Nigel J.	Cairns		PhD, FRCPath				
Erin	Franklin		MS, CCRP				
Lisa	Taylor-Reinwald		BA, HTL				

Supplemental Online Content: Nonauthor Collaborators

*Indicates required information. Only first name, last name, and suffix will appear in PubMed.

First Name, Middle Initial*	Last Name*	Suffix*	Academic Degrees	Institution	Location (city, state/province, country)	Role or Contribution, eg, chair, principal investigator	Subgroup, eg, Steering Committee
Virginia	Lee		PhD, MBA				
Magdalena	Korecka		PhD				
Michal	Figurski		PhD				
Karen	Crawford						
Scott	Neu		PhD				
Tatiana M.	Foroud		PhD				
Steven	Potkin		MD UC				
Li	Shen		PhD				
Kelley	Faber		MS, CCRC				
Sungeun	Kim		PhD				
Kwangsik	Nho		PhD				
Lean	Thal		MD				
Neil	Buckholtz						
William	Potter		MD				
Marilyn	Albert		PhD				
Richard	Frank		MD, PhD				
John	Hsiao		MD				
